# High-Order Sequential Simulation via Statistical Learning in Reproducing Kernel Hilbert Space

**DOI:** 10.1007/s11004-019-09843-3

**Published:** 2019-12-07

**Authors:** Lingqing Yao, Roussos Dimitrakopoulos, Michel Gamache

**Affiliations:** 1 Department of Mathematics and Industrial Engineering, Polytechnique Montréal, Montreal, QC H3T 1J4 Canada; 2 COSMO – Stochastic Mine Planning Laboratory, Department of Mining and Materials Engineering, McGill University, 3450 University Street, Montreal, QC H3A 2A7 Canada

**Keywords:** Stochastic simulation, High-order spatial statistics, Statistical learning, Reproducing kernel, Multipoint simulation

## Abstract

The present work proposes a new high-order simulation framework based on statistical learning. The training data consist of the sample data together with a training image, and the learning target is the underlying random field model of spatial attributes of interest. The learning process attempts to find a model with expected high-order spatial statistics that coincide with those observed in the available data, while the learning problem is approached within the statistical learning framework in a reproducing kernel Hilbert space (RKHS). More specifically, the required RKHS is constructed via a spatial Legendre moment (SLM) reproducing kernel that systematically incorporates the high-order spatial statistics. The target distributions of the random field are mapped into the SLM-RKHS to start the learning process, where solutions of the random field model amount to solving a quadratic programming problem. Case studies with a known data set in different initial settings show that sequential simulation under the new framework reproduces the high-order spatial statistics of the available data and resolves the potential conflicts between the training image and the sample data. This is due to the characteristics of the spatial Legendre moment kernel and the generalization capability of the proposed statistical learning framework. A three-dimensional case study at a gold deposit shows practical aspects of the proposed method in real-life applications.

## Introduction

Stochastic simulations are used to quantify the spatial uncertainty in earth science or engineering applications. Since the early 1990s, the so-termed multipoint statistical simulation (MPS) methods (Guardiano and Srivastava 1993; Journel and Zhang 2006; Strebelle 2002) were first proposed to overcome the limitation of the second-order simulation approaches in reproducing the complex spatial patterns encountered in natural phenomena. Instead of using a theoretical variogram/covariance model, as is the case with conventional two-point geostatistical simulations, the MPS methods consider that the so-called training image (TI) contains the prior information of the spatial statistics or patterns of the attribute to be simulated. A spatial template is defined as a geometrical configuration of the relative locations among the multiple points, regardless of the coordinates. The known data within the spatial template at a certain location on the simulation grid acts as the conditioning data in the simulation and is termed a data event. Over the past decade, several state-of-the-art MPS algorithms have been proposed to improve the efficiency and reproduction of the curvilinear features (Mariethoz and Caers 2014; Remy et al. 2009).

An inherent limitation of the MPS algorithms is that the high-order spatial statistics of the available data are not systematically considered and are partly integrated in ad-hoc ways. This issue becomes more prominent when the spatial statistics of the TI and the sample data are different, leading to realizations conflicting with the spatial statistics of the sample data, especially when the latter data is relatively dense as is the case in mining applications (Goodfellow et al. 2012; Osterholt and Dimitrakopoulos 2007). As an alternative, high-order simulation methods are proposed to model a random field without any presumption of its probability distribution, and high-order spatial statistics are systematically incorporated in the model (Dimitrakopoulos et al. 2010; Mustapha and Dimitrakopoulos 2010a, b, 2011). The first algorithm of high-order simulation, HOSIM, approximates the probability density function (PDF) by the Legendre polynomial series through the so-called spatial cumulants (Dimitrakopoulos et al. 2010; Mustapha and Dimitrakopoulos 2010b, 2011). Further developments of the high-order simulation paradigm include the simulation of spatially correlated variables (Minniakhmetov and Dimitrakopoulos 2017) and the direct simulation at the block scale (de Carvalho et al. 2019). Most recently, Yao et al. (2018) proposed a new computational model of high-order simulation as a unified empirical function, which avoids CPU-demanding computations of expansion coefficients. Furthermore, a kernel function can be derived from this model and will be used in the present work.

A common issue that runs across all of the above-mentioned high-order simulation methods is that the approximation of the PDF by orthogonal polynomials cannot be guaranteed to be positive. The sensitivity of high-order polynomials to the rounding errors near the endpoints of the approximation weakens the convergence of polynomial series to a stable analytic function, as discussed in Minniakhmetov et al. (2018), who propose an approximation of the PDF using Legendre-like orthogonal splines as the basis functions, resulting in a significant improvement in numerical stability. As the deviation of the empirical statistics from the true expectation arises due to possible statistical conflicts between the sample data and the TI, the convergence of the approximation to the actual underlying PDF could be undermined. Under such a circumstance, a postprocessing step has to be introduced to correct the approximation. For example, the correction procedure through interpolation around the points of negative densities is applied in Mustapha and Dimitrakopoulos (2010b).

The present work proposes a new high-order simulation framework based on statistical learning (Vapnik 1995, 1998), which deliberately mitigates the statistical conflicts between the sample data and the TI, and also overcomes the limitation of approximating the PDF with the orthogonal expansion series. Statistical learning theory (Vapnik 1998) develops a new learning paradigm to explore functional dependency from a given data set without relying on prior knowledge, which contrasts with the classical statistical methods that are based on parametric models. According to the learning paradigm, a target model needs to be learned from the available data set, which represents the training data. The so-called learning machine (Vapnik 1998) is frequently given as a set of functions, from which a specific learning model is selected to approximate the target model according to certain criteria.

To interpret high-order simulation in terms of statistical learning, the training data are regarded as the available data from the sample data and/or the TI. The target model is the probability distribution related to the random field of the spatial attributes. The learning model is the approximated PDF of the target probability distribution, from which the realizations can be generated. The learning process for high-order simulation is driven by matching the expected high-order spatial statistics of the target probability distribution to the high-order spatial statistics observed from the available data. The matching of the high-order spatial statistics is the most challenging part and is approached herein by a learning process in a reproducing kernel Hilbert space (RKHS) (Scholkopf and Smola 2001). A spatial Legendre moment (SLM) reproducing kernel is proposed to construct the specified RKHS (SLM-RKHS), such that the high-order spatial statistics are systematically incorporated in this Hilbert space for a certain probability distribution. The elements in the original data space are mapped into the SLM-RKHS, termed RKHS embedding (Muandet et al. 2016; Smola et al. 2007; Song et al. 2008, 2013). In addition, the high-order spatial statistics of the available data are carried over to the domain after this RKHS embedding. Eventually, the statistical learning regarding high-order simulation leads to a convex optimization in SLM-RKHS where the solutions amount to solving a quadratic programming problem.

In the following sections, the general theory of kernel methods, including the RKHS and RKHS embedding of probability distributions, are introduced. Section 3 describes the main workflow of high-order sequential simulation via statistical learning, and a spatial Legendre moment reproducing kernel is defined to construct the specific SLM-RKHS. Furthermore, this SLM-RKHS is decomposed to lower-dimensional subspaces, such that conditional probability density functions (CPDF) in the context of sequential simulation can be embedded into the corresponding subspaces. Subsequently, a high-order stochastic simulation method is presented as a learning process based on the embedding of the CPDF into the decomposed subspace of the SLM-RKHS. Next, the proposed simulation method is tested using a fully known data set. A case study at a gold deposit is then presented to show the practical aspects of the proposed method. Conclusions follow.

## Methods

### Overview of Kernel Space and Embedding a Probability Distribution

In the general setting of kernel methods, a kernel space needs to be set up and associated with a predefined kernel function, and a feature mapping is defined to map an arbitrary element from the original data space into the kernel space. The related general concepts and theory are formalized in the followed subsections.

#### Reproducing Kernel Hilbert Space

A Hilbert space $\mathcal{H}$ is a vector space over a field endowed with an inner product (Stein and Shakarchi 2005). For simplicity, the Hilbert space $\mathcal{H}$ over the set ${\mathbb{R}}$ of real numbers is considered here, and the inner product is defined as

$$ \left\langle {f,{\text{g}}} \right\rangle {:}\,{\mathcal{H}} \times {\mathcal{H}} \to {\mathbb{R}},\quad \forall f,{ }g \in {\mathcal{H}}. $$

The norm is defined as

$$ \left\| f \right\|_{{\mathcal{H}}} = \left\langle {f,f} \right\rangle^{{1/2{ }}} ,\quad { }\forall f \in {\mathcal{H}}. $$

Other essential properties can be found in Stein and Shakarchi (2005). The concepts of reproducing kernel and positive definite function are from Berlinet and Thomas-Agnan (2004) with the modification of the range of kernel function to ${\mathbb{R}}$.

##### Reproducing Kernel

Let ${\mathbb{E}}$ be a non-empty set and $\mathcal{H}$ be a Hilbert space of functions defined on ${\mathbb{E}}$. Then, a function $K{:}\,{\mathbb{E}}\times {\mathbb{E}}\to {\mathbb{R}}$ is a reproducing kernel of a Hilbert space $\mathcal{H}$ if and only if

1. $$\forall t\in {\mathbb{E}}, K(\cdot ,t)\in \mathcal{H}$$, and
2. $$\forall t\in {\mathbb{E}}, \forall f\in \mathcal{H},\langle f,K\left(\cdot ,t\right)\rangle =f(t)$$.

The last condition is called “the reproducing property,” because any function in $\mathcal{H}$ can be reproduced by its inner product with the kernel $K$. In addition, as a direct derivation of the above conditions, the reproducing kernel can be written as the inner product

$$ K\left( {s,t} \right) = \left\langle {K\left( { \cdot ,s} \right),{ }K\left( { \cdot ,{ }t} \right)} \right\rangle ,\quad \forall s,{ }t \in {\mathbb{E}}. $$

Naturally, a Hilbert space in possession of a reproducing kernel is called a reproducing kernel Hilbert space. The feature map associated with an RKHS $\mathcal{H}$ with kernel $K$ is defined as $\phi{:}\,{\mathbb{E}}\to \mathcal{H}$ such that $\langle \phi \left(s\right), \phi \left(t\right)\rangle =K(s, t)$. In fact, $\phi \left(t\right){:}\,{\mathbb{E}}\to \mathcal{H},t\mapsto K\left(\cdot , t\right),\forall t\in {\mathbb{E}}$ satisfies such a definition as the feature map according to the reproducing property. This type of feature map is called a reproducing kernel map (Scholkopf and Smola 2001) or canonical feature map (Steinwart and Christmann 2008) and will be used in the present paper.

##### Positive Definite Function

A real-valued function $K{:}\,{\mathbb{E}}\times {\mathbb{E}}\to {\mathbb{R}}$ is positive definite if $\forall n\ge 1, \forall \left({a}_{1},\dots ,{a}_{n}\right)\in {\mathbb{R}}^{n}, \forall ({x}_{1},\dots ,{x}_{n})\in {\mathbb{E}}^{n}$, there is

$$ \mathop \sum \limits_{i = 1}^{n} \mathop \sum \limits_{j = 1}^{n} a_{i} a_{j} K\left( {x_{i} ,x_{j} } \right) \ge 0 $$

The reason for introducing the concept of the positive definite function is that a reproducing kernel is equivalently a positive definite function (Berlinet and Thomas-Agnan 2004). Thus, in practical terms, constructing an RKHS is equivalent to defining a positive definite function.

#### RKHS Embedding of a Probability Distribution

The range of the feature mapping spans RKHS $\mathcal{H}$ by definition (Scholkopf and Smola 2001). Thus, the feature mapping $\phi$ is crucial in embedding a data element into the RKHS $\mathcal{H}$. Accordingly, two mappings are important to embed a probability distribution into the RKHS $\mathcal{H}$ (Smola et al. 2007)

1 $$ \begin{array}{*{20}c} {\mu \left[ p \right] = {{E}}_{x\sim p} \left[ {\phi \left( x \right)} \right],} \\ \end{array} $$

and

2 $$ \begin{array}{*{20}c} {\mu \left[ X \right] = \frac{{1}}{M}\mathop \sum \limits_{i = 1}^{M} \phi \left( {X_{i} } \right),} \\ \end{array} $$

where the first equation is the expectation kernel mean map regarding the density $p$ and the second one is the empirical kernel mean map with the finite sample set $X=\{{X}_{1},\dots ,{X}_{M}\}$. The expectation kernel mean map $\mu [p]$ is an element in the RKHS $\mathcal{H}$ as long as ${{E}}_{x\sim p}\left[K\left(x,x\right)\right]<\infty$ (Smola et al. 2007). Suppose that the samples from $X$ are independently drawn from the same probability distribution with density $p$, then $\mu [p]$ can be approximated by $\mu [X]$ (Song et al. 2008), with the bound of the deviation ${\Vert \mu \left[p\right]-\mu \left[X\right]\Vert }_{\mathcal{H}}$ with the probability given by Altun and Smola (2006).

The space of all probability distributions forms a convex set$\mathcal{P}$; thus, the image of the expectation kernel mean map $\mathcal{M}:=\{\mu \left[p\right], \forall p\in \mathcal{P}\}$ is also convex and is called the marginal polytope (Smola et al. 2007). In terms of the RKHS embedding, the goal of the density estimation is to find an optimal probability density $\widehat{p}\in \mathcal{P}$ such that the deviation ${\Vert \mu \left[X\right]-\mu \left[\widehat{p}\right]\Vert }_{\mathcal{H}}$ is minimized. In practice, the density estimator $\widehat{p}$ is assumed as a mixture of a set of candidate densities or prototypes ${p}_{i}$ (Smola et al. 2007; Song et al. 2008) as

3 $$ \begin{array}{*{20}c} {\hat{p} = \mathop \sum \limits_{i = 1}^{n} \alpha_{i} p_{i} ,} \\ \end{array} $$

where $$\sum\nolimits_{i}^{n} {\alpha_{i} = 1}$$ and $$\alpha_{i} \ge 0, \forall 1 \le i \le n$$.

Let us define the subset ${\mathcal{P}}_{0}$ of ${\mathcal{P}}$ as

$$ {\mathcal{P}}_{0} : = \left\{ {\hat{p} = \mathop \sum \limits_{i}^{n} \alpha_{i} p_{i} |\mathop \sum \limits_{i = 1}^{n} \alpha_{i} = 1{\text{ and }}\alpha_{i} \ge 0,\quad \forall 1 \le i \le n} \right\}.{ } $$

It can be seen that ${\mathcal{P}}_{0}$ is a convex hull of the prototypes since $\widehat{p}$ is a convex combination of the candidate densities. The density estimation amounts to solving the minimization problem restricted to a convex set ${\mathcal{P}}_{0}$ as

4 $$ \begin{array}{*{20}c} {\mathop {\min }\limits_{{\hat{p} \in {\mathcal{P}}_{0} }} \left\| {\mu \left[ X \right] - \mu \left[ {\hat{p}} \right]} \right\|_{{\mathcal{H}}}^{2} .} \\ \end{array} $$

Explicit expansion of Eq. (4) leads to solving a quadratic program for $\boldsymbol{\alpha }=({\alpha }_{1},\dots ,{\alpha }_{n})$ as the following (Song et al. 2008)

5 $$ \begin{array}{*{20}l} {} \hfill & {\mathop {\min }\limits_{{{\varvec{\alpha}} }} \frac{{1}}{2}{\varvec{\alpha}}^{T} \left( {{\mathbf{Q}} + \lambda {\mathbf{I}}} \right)\alpha - {\mathbf{q}}^{T} \alpha } \hfill \\ {{\text{s.t.}}} \hfill & {\mathop \sum \limits_{i = 1}^{n} \alpha_{i} = 1} \hfill \\ {} \hfill & {\alpha_{i} \ge 0,\quad \forall 1 \le i \le n} \hfill \\ \end{array} , $$

where $\lambda$ is a regularization constant to prevent overfitting, and $\mathbf{I}$ is the identity matrix. $\mathbf{Q}={\left[{Q}_{ij}\right]}_{n\times n}$ is a matrix, and $\mathbf{q}=({q}_{1},\dots ,{q}_{n})$ is a vector of length $n$, both of which are entries that depend on the kernel function. The matrix $\mathbf{Q}$ is positive definite; hence the above quadratic program (5) is a convex optimization problem.

### High-Order Simulation Method in Spatial Legendre Moment Kernel Space

#### SLM Reproducing Kernel

The motivation for applying statistical learning to the high-order simulation is to match the high-order spatial statistics of the output realizations to the training data through the learning process. This goal is achieved by the learning procedure in a newly defined kernel space, while the kernel is defined as

6 $$ \begin{array}{*{20}c} {K\left( {{\varvec{X}},{\varvec{Y}}} \right) = \mathop \prod \limits_{i = 0}^{N} \left[ {\mathop \sum \limits_{w = 0}^{W} \left( {w + \frac{{1}}{2}} \right)P_{w} \left( {x_{i} } \right)P_{w} \left( {y_{i} } \right)} \right],} \\ \end{array} $$

and is called a spatial Legendre moment kernel (SLM-kernel for short) of order *W*, where ${\varvec{X}},{\varvec{Y}}\in {[-\,1, 1]}^{N+1}, {\varvec{X}}=\left({x}_{0},{x}_{1},\dots ,{x}_{N}\right), {\varvec{Y}}=\left({y}_{0},{y}_{1},\dots ,{y}_{N}\right)$, and ${P}_{w}\left(\cdot \right)$ is the Legendre polynomial of order $w$ defined on the interval $[-\,1, 1]$.

As the name of the kernel suggests, one reason to define the SLM-kernel in the form of Eq. (6) is that past studies of high-order simulations based on Legendre-polynomial series have shown the capacity for capturing complex spatial patterns with spatial cumulants or spatial Legendre moments (Dimitrakopoulos et al. 2010; Mustapha and Dimitrakopoulos 2010b; Yao et al. 2018). In other words, the SLM-kernel is constructed in a way that the distance between two distributions embedded into the kernel space actually represent the deviation of spatial Legendre moments from each other. The other reason stems from the fact that the computational model from Yao et al. (2018) leads to a kernel-like expression of approximating the CPDF [cf. Eq. (14) in Yao et al. (2018)].

To prove that $K({\varvec{X}}, {\varvec{Y}})$ is positive definite, one can first define a simpler function $k\left(s, t\right)={P}_{w}\left(s\right){P}_{w}\left(t\right), \forall s, t\in [-\,1, 1]$ and show that it is positive definite. In fact,

$$ \forall n \ge 1,\quad \forall a_{i} ,a_{j} \in {\mathbb{R}},\quad \forall t_{i} \in \left[ { - 1,{ }1} \right],\quad { }1 \le i,j \le n, $$

it is easy to see that

$$ \mathop \sum \limits_{i = 1}^{n} \mathop \sum \limits_{j = 1}^{n} a_{i} a_{j} P_{w} \left( {t_{i} } \right)P_{w} \left( {t_{j} } \right) = \left[ {\mathop \sum \limits_{i = 1}^{n} a_{i} P_{w} \left( {t_{i} } \right)} \right]^{2} \ge 0 $$

Therefore, $k\left( {s,t} \right)$ is positive definite. Now, we denote

$$ \begin{aligned} K^{{\prime }} \left( {{\varvec{X}},{\varvec{Y}}} \right) & = \mathop \sum \limits_{w = 0}^{W} \left( {w + \frac{{1}}{2}} \right)P_{w} \left( {x_{i} } \right)P_{w} \left( {y_{i} } \right) \\ & = \mathop \sum \limits_{w = 0}^{W} \left( {w + \frac{{1}}{2}} \right)k\left( {x_{i} ,y_{i} } \right) \\ \end{aligned}. $$

${K}^{{\prime}}\left({\varvec{X}}, {\varvec{Y}}\right)$ is positive definite because the weighted sum of positive definite functions with non-negative coefficients is also positive definite. Finally, $K\left({\varvec{X}},{\varvec{Y}}\right)$ can be written as $K\left({\varvec{X}}, {\varvec{Y}}\right)={\prod }_{i=0}^{N}{K}^{{\prime}}\left({\varvec{X}}, {\varvec{Y}}\right)$. Given that the finite product of positive definite functions is also positive definite (Steinwart and Christmann 2008), it is proven that the function $K({\varvec{X}}, {\varvec{Y}})$ is positive definite, and thus, it defines a reproducing kernel.

#### Sequential Simulation via Statistical Learning in SLM-Kernel Space

The implementation of a high-order stochastic simulation is under the framework of a sequential simulation (Journel 1994). By means of decomposing the multivariate probability distribution into a consecutive set of univariate distributions, the simulation is carried out sequentially to generate random values from conditional distributions per a random path visiting the simulation grid. Specifically, let us denote the random field to be simulated as ${\varvec{Z}}({\varvec{u}})$, which composes a multivariate distribution regarding the variable locations ${\varvec{u}}$ at a discrete simulation grid. Suppose an arbitrary node ${Z}_{0}$ to be simulated within a random path is located at ${{\varvec{u}}}_{0}$ with a neighborhood $\Lambda$ of $N$ conditioning data that contains either the sample data or the previously simulated nodes along the random path. Without loss of generality, the key problem in the stochastic simulation is to find an estimation of the CPDF $f({Z}_{0}|\Lambda )$, given the center node ${Z}_{0}$ and the $N$ conditioning data. From the spatial configuration of the neighborhood, a spatial template can be extracted as ${\varvec{T}}=({{\varvec{u}}}_{0},{{\varvec{u}}}_{0}+{h}_{1},\dots , {{\varvec{u}}}_{0}+{h}_{N})$, where ${h}_{1},\dots ,{h}_{N}$ are distance vectors of the location of each conditioning data from the center node ${u}_{0}$.

Clearly, statistical learning for the simulation aims to learn a target probability distribution from the available training data, and this turns out to be minimizing the distance of the empirical distribution and the target distribution after embedding them into the SLM-kernel space. By the definition of the Dirac delta function, one can define an empirical probability density function (EPDF) (Scott 2015) corresponding to a sample set $X$ of size $M$ as

7 $$ \begin{array}{*{20}c} {f_{{{\text{emp}}}} \left( x \right) = \frac{{1}}{M}\mathop \sum \limits_{i = 1}^{M} \delta \left( {x - X_{i} } \right).} \\ \end{array} $$

Then, the empirical kernel mean map $\mu \left[ X \right]$ can be rewritten as a convolution with the kernel $K$ as

8 $$ \begin{array}{*{20}c} {\mu_{K} \left[ {f_{{{\text{emp}}}} } \right] : = \mu \left[ X \right] = \int {f_{{{\text{emp}}}} } \left( x \right)K\left( {x, \cdot } \right){\text{d}}x.} \\ \end{array} $$

Similarly, the expectation kernel mean map $\mu \left[ p \right]$ can also be written as

9 $$ \begin{array}{*{20}c} {\mu_{K} \left[ p \right] = \int p \left( x \right)K\left( {x, \cdot } \right){\text{d}}x. } \\ \end{array} $$

In this way, both the empirical kernel mean map $\mu [X]$ and the expectation kernel mean map $\mu [p]$ can be regarded as an integral operator ${\mu }_{K}$ determined by the kernel $K$ acting on the EPDF or the PDF. The convolution of the density function with kernels can be analogous to the regularization of the integral operator to solve the ill-posed problem of density estimation (Vapnik 1998; Vapnik and Mukherjee 1999).

Given the above-mentioned template ${\varvec{T}}=({{\varvec{u}}}_{0},{{\varvec{u}}}_{0}+{h}_{1},\dots , {{\varvec{u}}}_{0}+{h}_{N})$ and the replicate encountered in the TI as ${{\varvec{\zeta}}}_{t}=({\zeta }_{t,0},{\zeta }_{t,1},\dots ,{\zeta }_{t,N})$ corresponding to ${\varvec{T}}$, the EPDF ${f}_{\mathrm{e}\mathrm{m}\mathrm{p}}$ embedded in the SLM-RKHS is identical to the density estimator in Yao et al. (2018) in the kernel form as

10 $$ \begin{array}{*{20}c} {\mu_{K} \left[ {f_{{{\text{emp}}}} } \right] = \frac{{1}}{M}\mathop \sum \limits_{t = 1}^{M} K\left( {{\varvec{\zeta}}_{t} ,{ } \cdot } \right).} \\ \end{array} $$

Furthermore, under the sequential simulation framework, the CPDF $f({Z}_{0}|\Lambda )$ can be mapped into a lower-dimensional kernel space through decomposition of the kernel space, so that the high-order simulation can be reduced to a one-dimensional optimization problem.

Note that the kernel $K$ in Eq. (6) can be decomposed as a product of lower-dimensional kernels ${K}_{0}$ and ${K}_{N}$ as

11 $$ \begin{array}{*{20}c} {K_{0} \left( {x_{0} ,y_{0} } \right) = \mathop \sum \limits_{w = 0}^{W} \left( {w + \frac{{1}}{2}} \right)P_{w} \left( {x_{0} } \right)P_{w} \left( {y_{0} } \right),} \\ \end{array} $$

and

12 $$ \begin{array}{*{20}c} {K_{N} \left( {{\varvec{X}}^{{\prime }} ,{\varvec{Y}}^{{\prime }} } \right) = \mathop \prod \limits_{i = 1}^{N} \left[ {\mathop \sum \limits_{w = 0}^{W} \left( {w + \frac{{1}}{2}} \right)P_{w} \left( {x_{i}^{{\prime}} } \right)P_{w} \left( {y_{i}^{{\prime}} } \right)} \right],} \\ \end{array} $$

where ${K}_{0}$ is one-dimensional and ${K}_{N}$ is $N$-dimensional with ${\varvec{X}}\boldsymbol{^{\prime}}=\left({x}_{1},\dots ,{x}_{N}\right), {\varvec{Y}}\boldsymbol{^{\prime}}=\left({y}_{1},\dots ,{y}_{N}\right)$. Through marginalization of Eq. (10), the approximation of the CPDF ${\tilde{f}}_{W}({z}_{0}|\Lambda )$ can be rewritten in terms of the kernels as

13 $$ \begin{array}{*{20}c} {\tilde{f}_{W} \left( {z_{0} {{|\Lambda }}} \right) = \frac{{\mathop \sum \nolimits_{t = 1}^{M} K_{0} \left( {\zeta_{t,0} ,z_{0} } \right) \cdot K_{N} \left( {{\varvec{\zeta}}_{t}^{{\prime}} ,{\Lambda }} \right)}}{{\mathop \sum \nolimits_{t = 1}^{M} K_{N} \left( {{\varvec{\zeta}}_{t}^{{\prime}} ,{\Lambda }} \right)}},} \\ \end{array} $$

where ${\varvec{\zeta}}_{t}^{{\prime}} = \left( {\zeta_{t,1} , \ldots ,\zeta_{t,N} } \right)$. By letting

14 $$ \begin{array}{*{20}c} {\beta_{t} = \frac{{K_{N} \left( {{\varvec{\zeta}}_{t}^{{\prime}} ,{\Lambda }} \right)}}{{\mathop \sum \nolimits_{t = 1}^{M} K_{N} \left( {{\varvec{\zeta}}_{t}^{{\prime}} ,{\Lambda }} \right)}},} \\ \end{array} $$

the approximation of the CPDF $\tilde{f}_{W} (z_{0} |{\Lambda })$ can be expressed as

15 $$ \begin{array}{*{20}c} {\tilde{f}_{W} \left( {z_{0} {{|\Lambda }}} \right) = \mathop \sum \limits_{t = 1}^{M} \beta_{t} \cdot K_{0} \left( {\zeta_{t,0} ,z_{0} } \right).} \\ \end{array} $$

From Eq. (15), it turns out that the approximated CPDF ${\tilde{f}}_{W}({z}_{0}|\Lambda )$ is a linear combination of kernel bases, and therefore, it lies in the SLM-RKHS with the kernel ${K}_{0}$. Furthermore, it can be regarded as the embedding of the empirical CPDF into the SLM-RKHS. In other words, the kernel mean map ${\mu }_{{K}_{0}}$ for the conditional distributions can be defined as

16 $$ \begin{array}{*{20}c} {\mu_{{K_{0} }} \left[ {f_{{{\text{emp}}}} \left( {z_{0} {{|\Lambda }}} \right)} \right] = \mathop \sum \limits_{t = 1}^{M} \beta_{t} \cdot K_{0} \left( {\zeta_{t,0} ,{ } \cdot } \right),} \\ \end{array} $$

and

17 $$ \begin{array}{*{20}c} {\mu_{{K_{0} }} \left[ {f\left( {z_{0} {{|\Lambda }}} \right)} \right] = \int f\left( {z_{0} {{|\Lambda }}} \right)K_{0} \left( {z_{0} ,{ } \cdot } \right){\text{d}}z_{0} = E\left[ {K_{0} \left( {z_{0} ,{ } \cdot } \right)} \right],} \\ \end{array} $$

where Eqs. (16) and (17) correspond to the SLM-RKHS embedding of the empirical CPDF and the target CPDF, respectively.

Assuming that the CPDF can be expressed as the convex combination of some candidate distributions ${p}_{i}$ as in Eq. (3), such that $f\left({z}_{0}|\Lambda \right)\in {\mathcal{P}}_{0}$, then the density estimation for the CPDF can be solved by a similar minimization problem as Eq. (4) with the kernel mean map changing to ${\mu }_{{K}_{0}}$. Explicit expansion of the minimization problem leads to a quadratic program similar to Eq. (5), whereas the matrix $\mathbf{Q}$ and the vector $\mathbf{q}$ are expressed as

18 $$ \begin{array}{*{20}c} {Q_{ij} = {{E}}_{{z_{0} \sim p_{i} ,{ }z_{0}^{{\prime}} \sim p_{j} }} \left[ {K_{0} \left( {z_{0} ,z_{0}^{{\prime}} } \right)} \right],} \\ \end{array} $$

19 $$ \begin{array}{*{20}c} {q_{j} = \mathop \sum \limits_{t = 1}^{M} \beta_{t} \cdot {{E}}_{{z_{0} \sim p_{j} }} \left[ {K_{0} \left( {\zeta_{t,0} ,z_{0} } \right)} \right].} \\ \end{array} $$

Therefore, combining Eqs. (5), (11), (18) and (19), the RKHS embedding of the CPDF leads to a quadratic program expressed by the one-dimensional kernel ${K}_{0}$. The solution to the optimization problem will give the weights ${\alpha }_{i}$ of each candidate distribution ${p}_{i},$ which leads to a target distribution matching to the high-order spatial statistics of the available data.

**Fig. 1:**
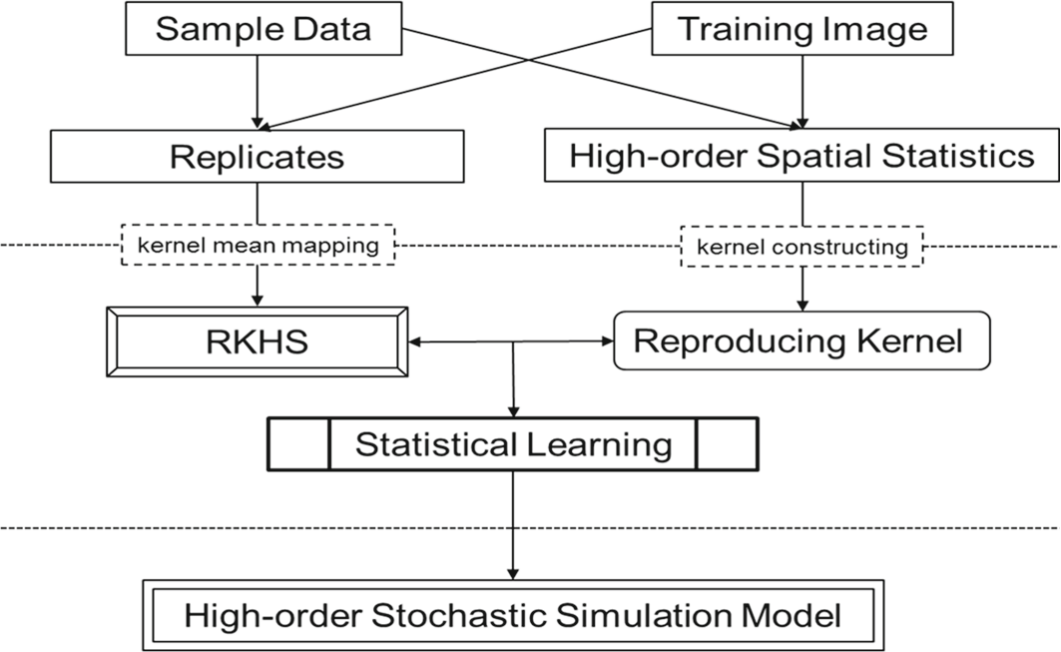
Workflow of high-order simulation via statistical learning

A general high-order stochastic simulation workflow via statistical learning is shown in Fig. 1. The main difference between the new high-order simulation workflow and the other geostatistical simulation methods is that the key element in the proposed workflow becomes the kernelization, including the kernel construction and the kernel mean mapping. A detailed implementation of the algorithm is given in Sect. 4.

## Sequential Simulation Algorithm Based on Statistical Learning in SLM-RKHS

The SLM-RKHS embedding of the CPDF projects the density estimation in high-order stochastic simulation into a quadratic program in the feature space with SLM-kernel ${K}_{0}$ defined in the interval $[-\,1, 1]$. Hence, the sample data and the TI are first transformed into the interval $[-\,1, 1]$. The truncated normal densities on the interval $[-\,1, 1]$ are used as the prototypes. Let us denote the normal density with mean ${m}_{i}$ and standard deviation $\sigma$ as ${g}_{{\sigma ,m}_{i}}$ and its corresponding cumulative distribution function as ${G}_{\sigma ,{m}_{i}}$. Then, the density functions of the prototypes are ${p}_{i}={g}_{\sigma ,{m}_{i}}/{c}_{i}$, with ${c}_{i}={G}_{\sigma ,{m}_{i}}\left(1\right)-{G}_{\sigma ,{m}_{i}}(-\,1)$. Thus, the approximated CPDF can be expressed as

20 $$ \begin{array}{*{20}c} {\hat{f}\left( {z_{0} {{|\Lambda }}} \right) = \mathop \sum \limits_{i = 1}^{n} \alpha_{i} g_{{\sigma ,m_{i} }} \left( {z_{0} } \right)/c_{i} ,} \\ \end{array} $$

where $n$ is the number of the prototypes. The computations of the matrix $\mathbf{Q}$ and the vector $\mathbf{q}$ are essential to build the quadratic program for solving the weights ${\alpha }_{i}$. Further expansions of ${Q}_{ij}$ and ${q}_{j}$ in Eqs. (18) and (19) give

21 $$ \begin{array}{*{20}c} {Q_{ij} = \mathop \sum \limits_{w = 0}^{W} \left( {w + \frac{{1}}{2}} \right)E_{{z_{0} \sim p_{i} }} \left[ {P_{w} \left( {z_{0} } \right)} \right] \cdot E_{{z_{0}^{{\prime}} \sim p_{j} }} \left[ {P_{w} \left( {z_{0}^{{\prime}} } \right)} \right],} \\ \end{array} $$

22 $$ \begin{array}{*{20}c} {q_{j} = \mathop \sum \limits_{t = 1}^{M} \beta_{t} \cdot \left( {\mathop \sum \limits_{w = 0}^{W} \left( {w + \frac{{1}}{2}} \right)P_{w} \left( {\zeta_{t,0} } \right)E_{{z_{0} \sim p_{j} }} \left[ {P_{w} \left( {z_{0} } \right)} \right]} \right).} \\ \end{array} $$

As the computations of the coefficients ${\beta }_{t}$ and the Legendre polynomial ${P}_{w}\left({\zeta }_{t,0}\right)$ are straightforward according to their definitions, the Legendre polynomial moment with the truncated normal density ${E}_{{z}_{0}\sim {p}_{i}}\left[{P}_{w}\left({z}_{0}\right)\right]$ remains the only term of more consideration. Here, a recursive algorithm to compute the Legendre polynomial moment ${E}_{{z}_{0}\sim {p}_{i}}\left[{P}_{w}\left({z}_{0}\right)\right]$ is developed.

Let us denote ${A}_{w,i}={E}_{{z}_{0}\sim {p}_{i}}\left[{P}_{w}\left({z}_{0}\right)\right]$ and ${B}_{w,i}= {E}_{{z}_{0}\sim {p}_{i}}\left[{z}_{0}{P}_{w}\left({z}_{0}\right)\right].$ Note that ${P}_{0}\left({z}_{0}\right)=1$, and ${P}_{1}\left({z}_{0}\right)={z}_{0}, \forall {z}_{0}\in [-\mathrm{1,1}]$. There are

23 $$ \begin{array}{*{20}c} {A_{0,i} = 1,} \\ \end{array} $$

and

24 $$ \begin{array}{*{20}c} {A_{1,i} = B_{0,i} = m_{i} + \sigma^{2} \left[ {g_{{\sigma ,{ }m_{i} }} \left( { - 1} \right) - g_{{\sigma ,{ }m_{i} }} \left( 1 \right)} \right]/c_{i} .} \\ \end{array} $$

The recursive relations of Legendre polynomials (Lebedev and Silverman 1965) are

25 $$ \begin{array}{*{20}c} {\left( {w + 1} \right)P_{w + 1} \left( {z_{0} } \right) = \left( {2w + 1} \right)z_{0} P_{w} \left( {z_{0} } \right) - wP_{w - 1} \left( {z_{0} } \right),} \\ \end{array} $$

and

26 $$ \begin{array}{*{20}c} {\left( {2w + 1} \right)P_{w} \left( {z_{0} } \right) = \frac{{\text{d}}}{{{\text{d}}z_{0} }}\left[ {P_{w + 1} \left( {z_{0} } \right) - P_{w - 1} \left( {z_{0} } \right)} \right].} \\ \end{array} $$

By Eqs. (25) and (26) and through integration by parts, one can derive the following recursive equations

27 $$ \begin{array}{*{20}c} {\left( {w + 1} \right)A_{w + 1,i} = \left( {2w + 1} \right)B_{w,i} - wA_{w - 1,i} ,} \\ \end{array} $$

and

28 $$ \begin{aligned} B_{w,i} & = m_{i} A_{w,i} + \sigma^{2} \left[ {\left( { - 1} \right)^{w} g_{{\sigma ,m_{i} }} \left( { - 1} \right) - g_{{\sigma ,m_{i} }} \left( 1 \right)} \right]/c_{i} \\ & \quad + \,\sigma^{2} \left[ {(2\left( {w - 1} \right) + 1} \right]A_{w - 1,i} + \sigma^{2} \left[ {2\left( {w - 3} \right) + 1} \right]A_{w - 3,i} + \cdots . \\ \end{aligned} $$

Combining with the initial conditions in Eqs. (23) and (24), Eqs. (27) and (28) form a complete recursive procedure to compute ${E}_{{z}_{0}\sim {p}_{i}}\left[{P}_{w}\left({z}_{0}\right)\right]$. The computations in turn build the quadratic program for density estimation of the conditional probability distribution in the simulation.

In a situation with high-dimensional space, the location parameters ${m}_{i}$ of the prototypes can be determined by clustering the available data. Here, since the density estimation problem is cast to the one-dimensional space by kernel decomposition, the locations of the prototypes are given by a set of peak points of the function from Eq. (15). Specifically, the interval $[-\,1, 1]$ is divided evenly into 100 subintervals, and the prototypes are selected from the subintervals which contain the peak points of the function Eq. (15). This heuristic approach to selecting prototypes further simplifies the quadratic program and makes the simulation feasible for implementation. The scale parameter $\sigma$ can be chosen by the method of stochastic gradient descent where the gradients can be derived from the recursive equations in Eqs. (27) and (28).

In summary, the high-order stochastic simulation algorithm based on RKHS embedding (KERNELSIM hereafter for simplification) can be described as follows:

1. Scale the property values of the samples and the TI to the interval [−1, 1].
2. Generate a random path to visit the simulation grid.
3. Pick one node from the random path to simulate, with the conditioning data taken from the neighborhood containing both the sample data and the previously simulated nodes.
4. Replicates are scanned from the TI according to the template defined by the spatial configuration of the conditioning data.
5. Compute the SLM-kernel moments to build the quadratic program.
6. Solve the quadratic program to estimate of the CPDF with regard to the center node. Draw a random value from the CPDF as the data value of the center node.
7. Repeat from step (3) until the simulation is completed.
8. Back transform the property values of the simulation from $[-\,1, 1]$ to the original data space.

In a practical implementation, step (5) can be simplified to precompute the Legendre polynomial moments for each prototype distribution, as well as the Legendre polynomial values of the replicates, and therefore the computations can be greatly reduced at the cost of more memory usage. The solver for the quadratic program in step (6) applied to the present paper is based on the algorithm from Goldfarb and Idnani (1983).

The time complexity of the proposed algorithm is of polynomial time overall. Suppose that the size of the simulation grid is $S$ and the size of the training data is $M$, the maximum order of the Legendre moments is $W$, the maximum number of conditioning data is $N$, and the number of the prototype distributions is ${n}_{p}$. Searching the replicates of the conditioning data from a regular grid takes $O(M\cdot N)$ operations. Computing the kernel moments and building the quadratic program takes $O\left(M\cdot {n}_{p}\left({W}^{3}+{W}^{2}N\right)\right)$ arithmetic operations. Solving the quadratic program problem also takes polynomial time of $O\left({n}_{p}^{4}\cdot L\right),$ where $L$ is the size of the problem encoding in binary (Vavasis 2001). Hence, the overall time complexity is a polynomial of $O\left(S\cdot (M\cdot {n}_{p}\left({W}^{3}+{W}^{2}N\right)+{n}_{p}^{4}\cdot L)\right)$.

## Case Studies

### Case Study at a Fully Known Reservoir

**Fig. 2:**
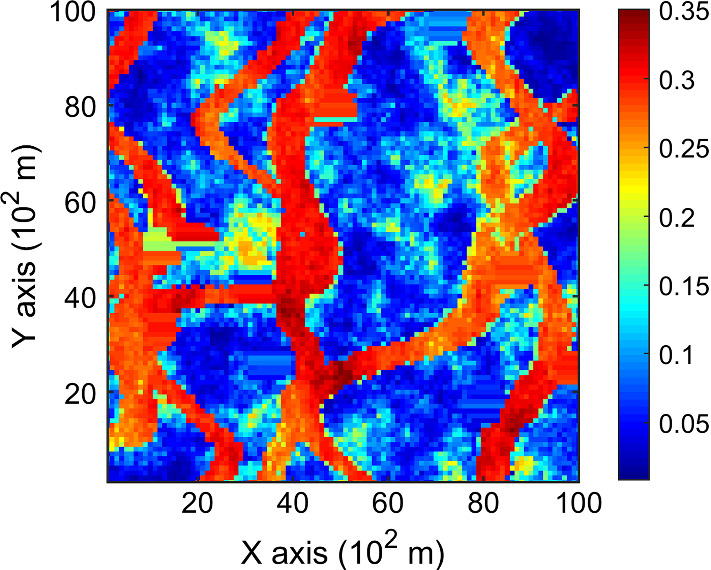
Exhaustive image: a horizontal section from a fully known reservoir

**Fig. 3:**
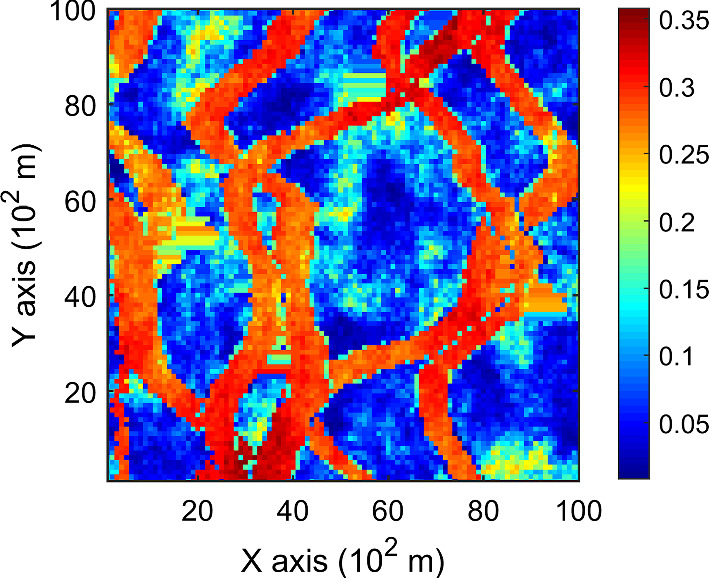
TI-1: another horizontal section from a fully known reservoir

**Fig. 4:**
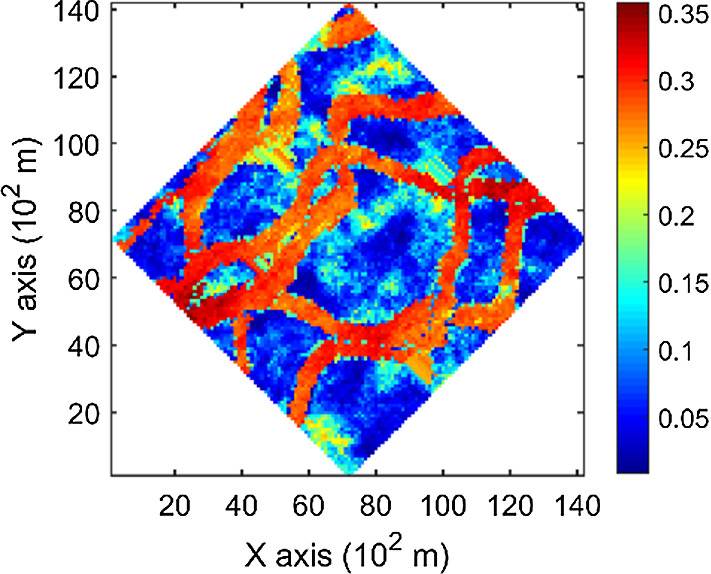
TI-2: rotation of TI-1 45° clockwise

**Fig. 5:**
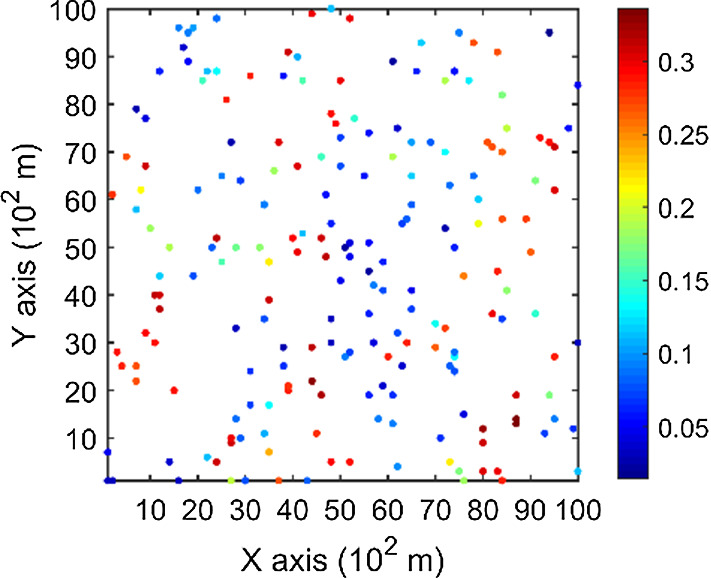
DS-1: data samples of 200 points drawn from the exhaustive image

**Fig. 6:**
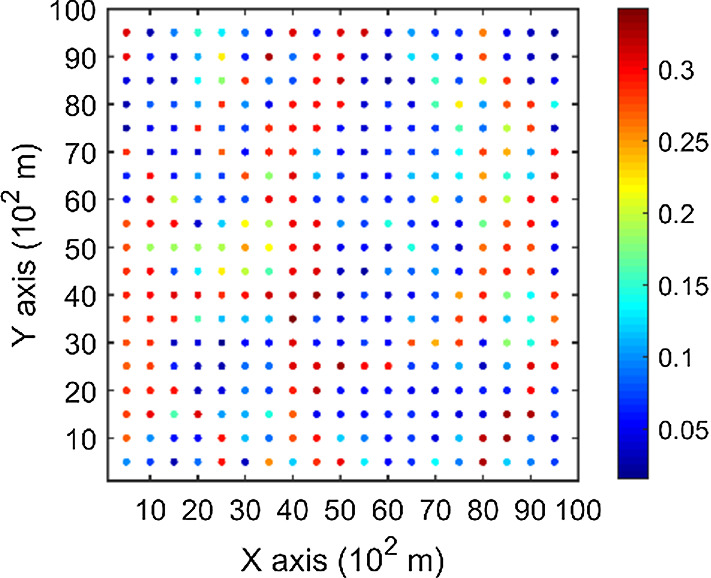
DS-2: data samples of 400 points drawn from the exhaustive image

The porosity attributes from the Stanford V reservoir data set (Mao and Journel 1999) are considered for simulation. Two horizontal sections at different depths are extracted from the reservoir, acting as the exhaustive image and the TI, respectively. For comparison, the two horizontal sections shown in Figs. 2 and 3 are selected to be the same ones used in a previous study (Yao et al. 2018). Firstly, the TI extracted from the original reservoir data set is rotated 45° clockwise to generate a new TI with seemingly different spatial structures, which are noted as TI-1 and TI-2 (Figs. 3, 4), respectively. Furthermore, two different sets of sample data as DS-1 and DS-2 are drawn from the exhaustive image and are shown in Figs. 5 and 6, corresponding to the relatively sparse and dense samples, respectively. The main purposes of performing a simulation on these different cases are: (1) testing the sensitivity of KERNELSIM to the statistical conflicts between the sample data and the TI; (2) testing the impact of the number of sample data on the realization of KERNELSIM.

#### Example 1

This example consists of simulation results generated by KERNELSIM with the TI-1 as the training image and DS-1 and DS-2 as the sample data sets. This example generally represents the situation where the sample data and the TI are of different origin but are sharing some similarity in spatial patterns. For instance, the channels in both the exhaustive image and TI-1 are preferential in the vertical directions.

**Fig. 7:**
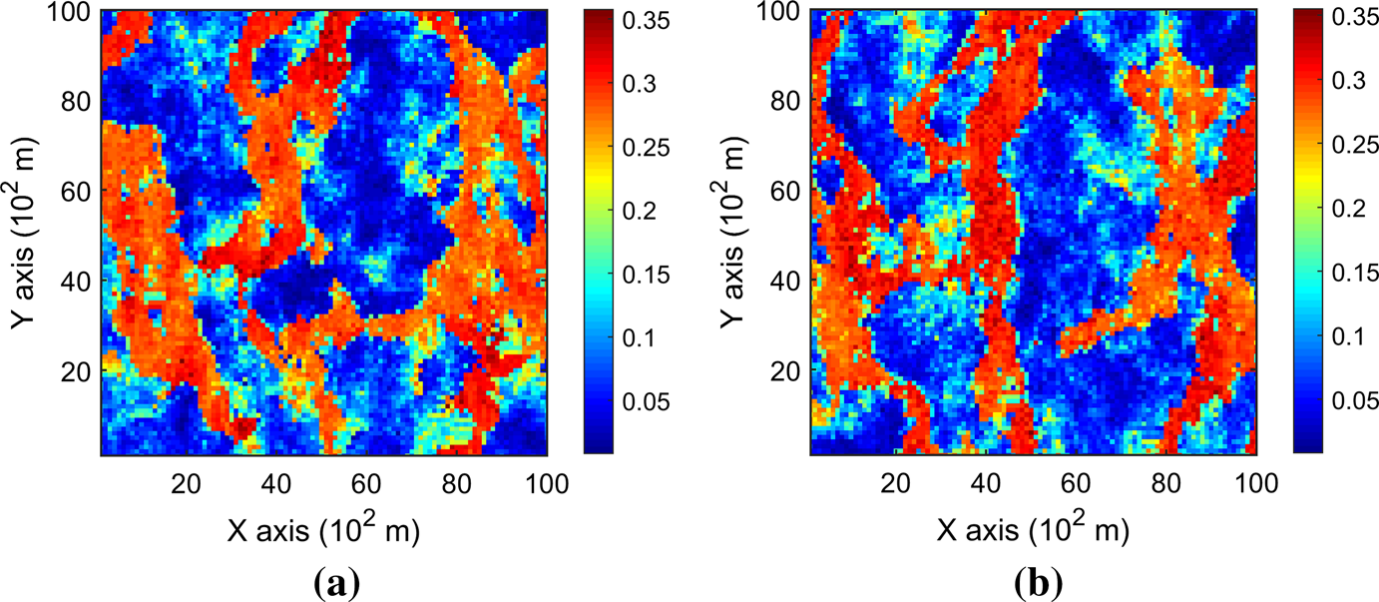
One realization from KERNELSIM using TI-1. **a** DS-1 as the sample data, **b** DS-2 as the sample data

**Fig. 8:**
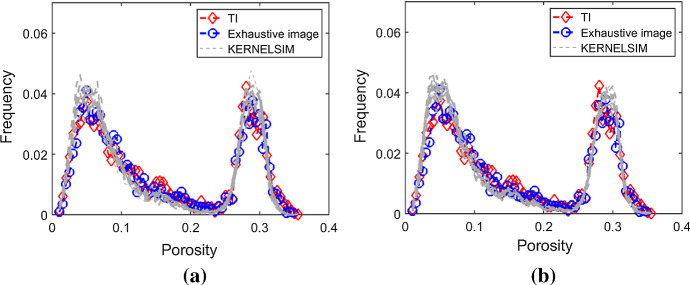
Histograms of 10 realizations of KERNELSIM using TI-1. **a** DS-1 as the sample data, **b** DS-2 as the sample data

**Fig. 9:**
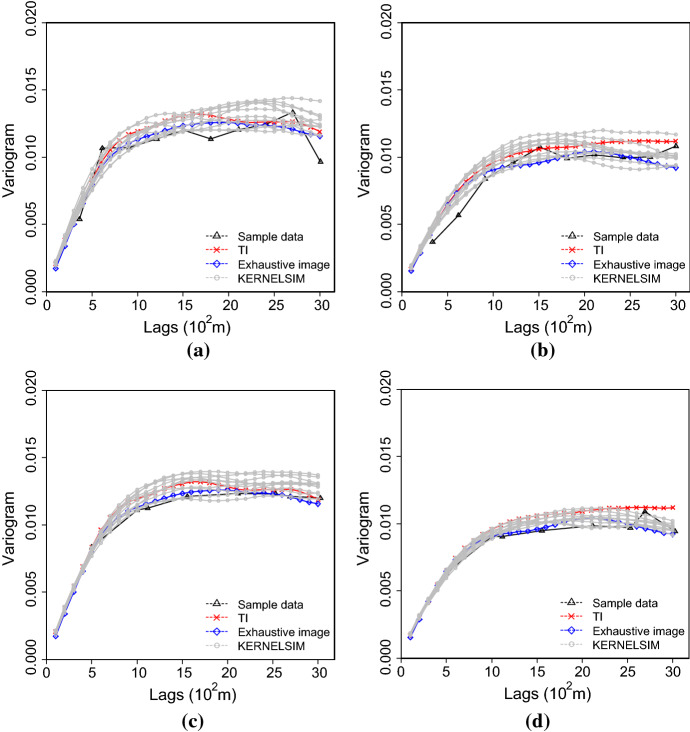
Variograms of 10 realizations of KERNELSIM using TI-1. **a**, **b** Along the *X* and *Y* axes with DS-1 as the sample data; **c**, **d**, along the *X* and *Y* axes with DS-2 as the sample data

**Fig. 10:**
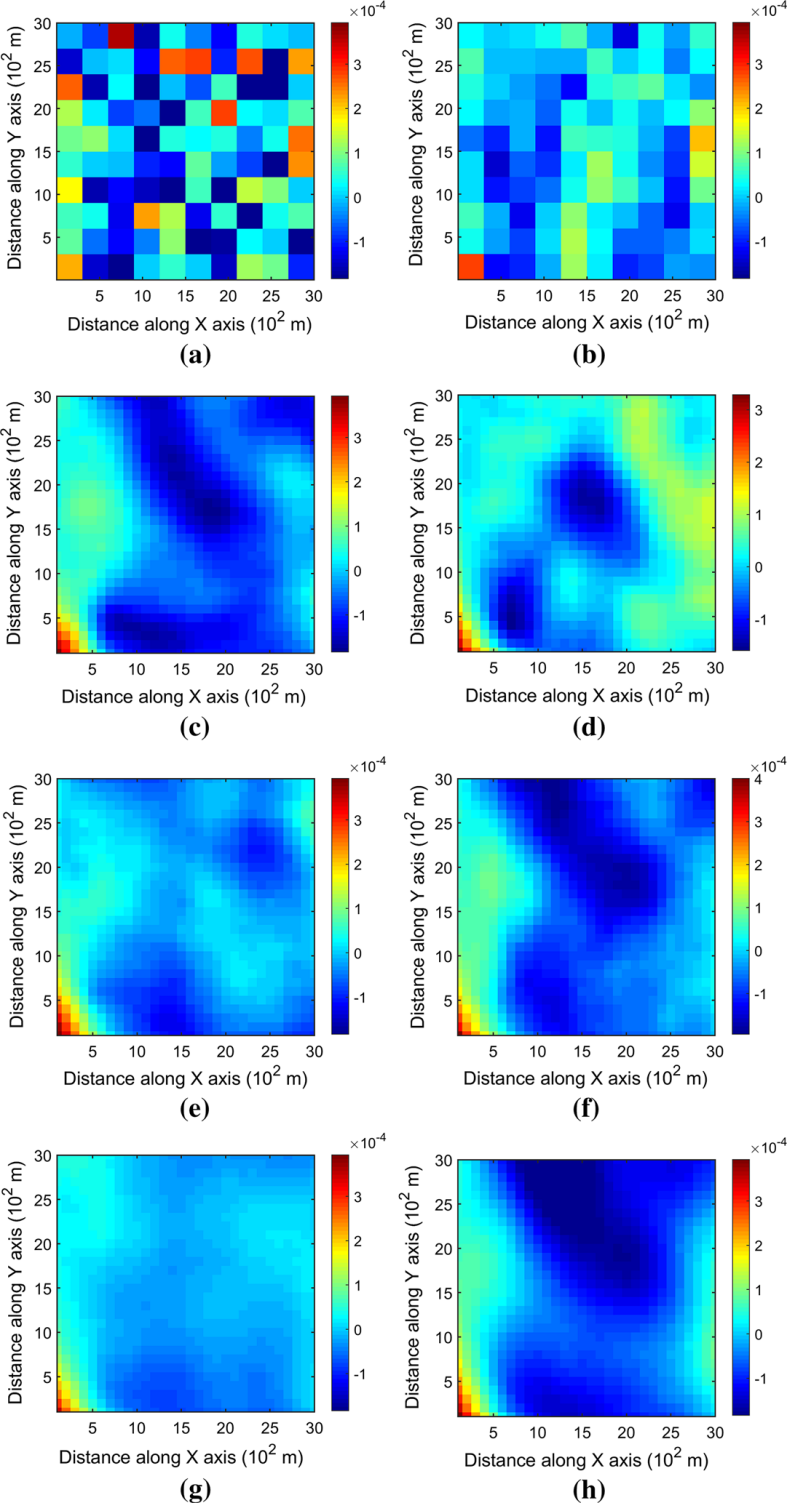
Third-order cumulant maps of **a** DS-1, **b** DS-2, **c** exhaustive image, **d** TI-1, **e** realization in Fig. 7a with DS-1 as the sample data, **f** realization in Fig. 7b with DS-2 as the sample data, **g** 10 realizations in average with DS-1 as the sample data, and **h** 10 realizations in average with DS-2 as the sample data

**Fig. 11:**
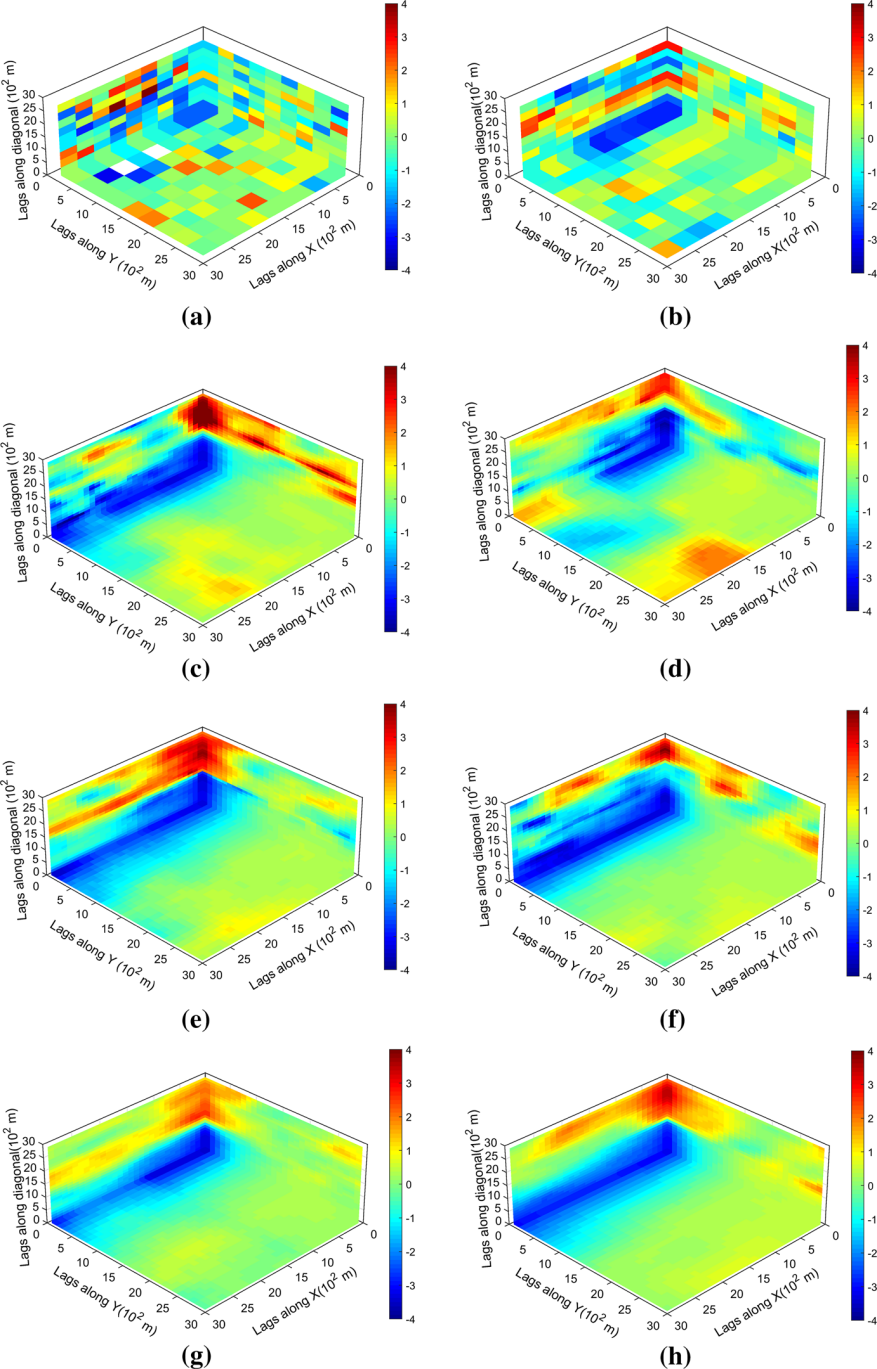
Fourth-order cumulant maps of **a** DS-1, **b** DS-2, **c** exhaustive image, **d** TI-1, **e** realization in Fig. 7a with DS-1 as the sample data, **f** realization in Fig. 7b with DS-2 as the sample data, **g** 10 realizations in average with DS-1 as the sample data, and **h** 10 realizations in average with DS-2 as the sample data

Figure 7 shows one realization of KERNELSIM using TI-1 as the training image and with DS-1 and DS-2 as the sample data, respectively. For comparison, both realizations are generated by the same random path to visit the nodes on the grid. It is clear that both realizations reproduce the main spatial structures of the exhaustive image along the vertical channels from the visualization (Fig. 7). The realization shown in Fig. 7a is comparable to the case study in Yao et al. (2018), and it shows that the present method reproduces channel connectivity better and eliminates the noisy points that appeared in the realizations generated using past approaches, which were caused by the impact of statistical conflicts between the sample data and the TI. Comparisons of the histograms and variograms of 10 realizations of KERNELSIM using either DS-1 or DS-2 as the sample data are illustrated in Figs. 8 and 9, respectively. The third-order cumulant maps of the sample sets DS-1 (smoothed for visualization) and DS-2 are shown in Fig. 10a, b. The cumulant maps of the exhaustive image and the TI are shown in Fig. 10c, d. For comparison, the third-order cumulant maps of the realizations of KERNELSIM using either the DS-1 or DS-2 as the sample data are shown in Fig. 10e, f. Figure 10g, h shows the average third-order cumulant maps of 10 realizations using the DS-1 and DS-2 as the sample data, respectively. Similarly, a further comparison of fourth-order cumulant maps is displayed in Fig. 11. The spatial template for computing the fourth-order cumulant maps included directions along the *X*-axis, *Y*-axis and the diagonal direction. The fourth-order cumulant maps are scaled by their deviations for clearer visualization of the patterns. Both the third-order and the fourth-order cumulant maps clearly show that the KERNELSIM realization tends to have similar spatial patterns to the sample data and the exhaustive image. The above results show that the KERNELSIM method reproduces both the lower and higher spatial statistics of the underlying random field given that the TI and the sample data share some similarity in their spatial distributions. Specifically, regardless the number of sample data being used, the main spatial features of the reservoir are retained in the realizations of KERNELSIM, as supported from the visual appearance of the vertical channels and the variograms, as well as from the cumulant maps.

#### Example 2

**Fig. 12:**
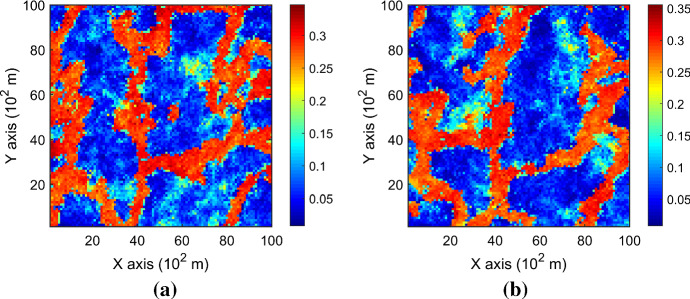
One realization from KERNELSIM using TI-2. **a** DS-1 as the sample data, **b** DS-2 as the sample data

**Fig. 13:**
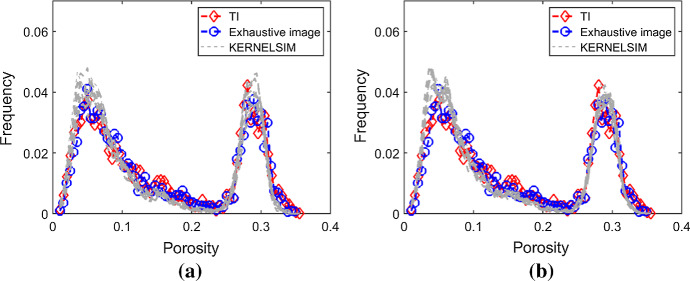
Histograms of 10 realizations of KERNELSIM using TI-2. **a** DS-1 as the sample data, **b** DS-2 as the sample data

**Fig. 14:**
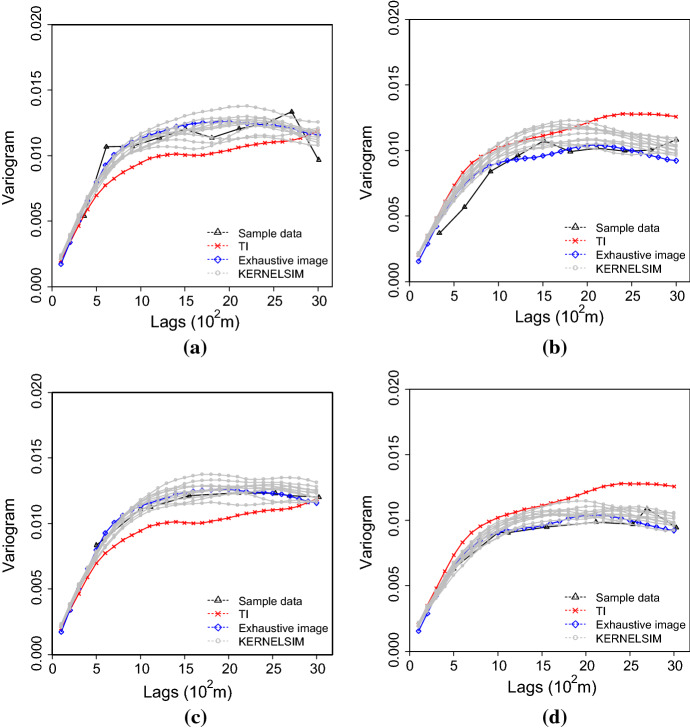
Variograms of 10 realizations of KERNELSIM using TI-2. **a**, **b** Along the *X* and *Y* axes with DS-1 as the sample data; **c**, **d**, along the *X* and *Y* axes with DS-2 as the sample data

**Fig. 15:**
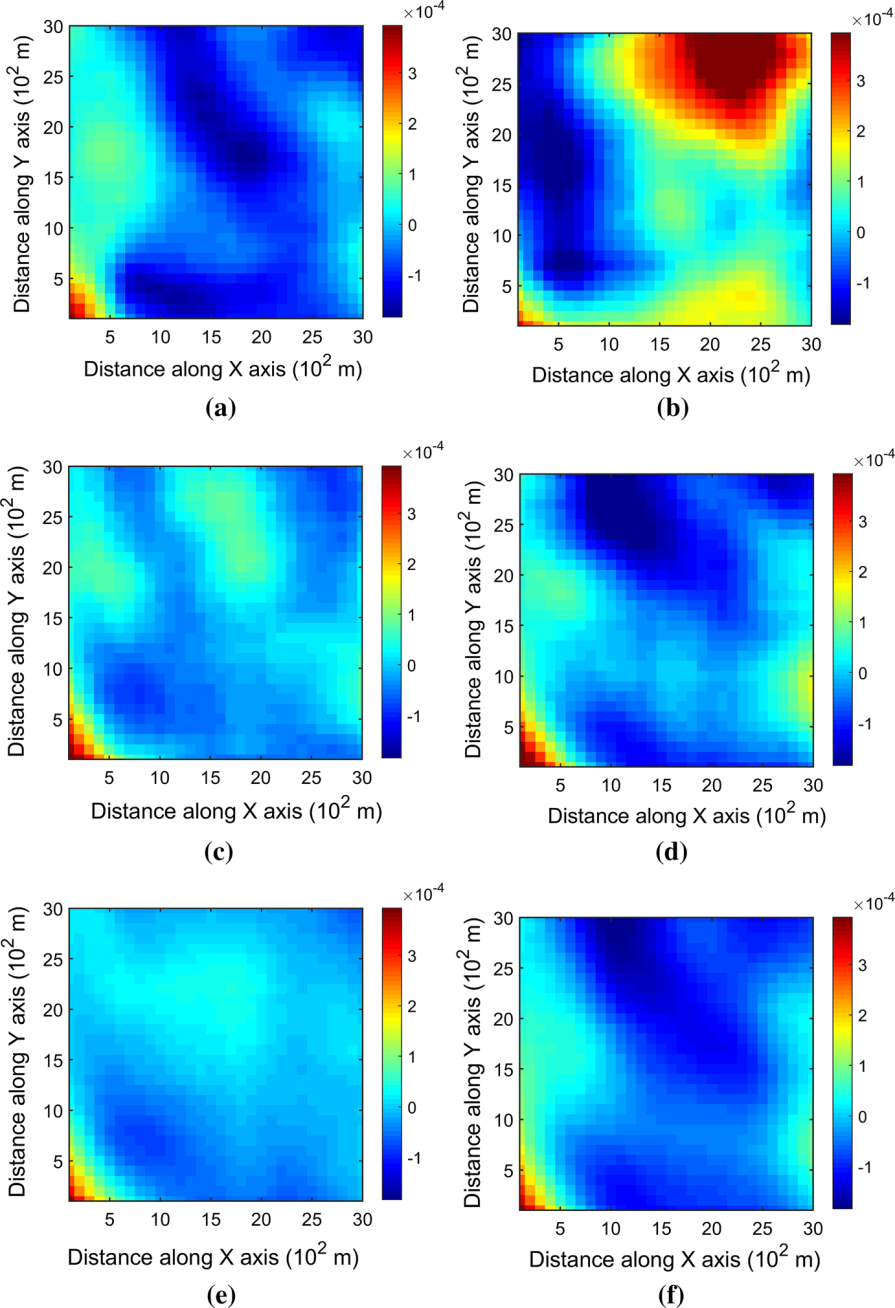
Third-order cumulant maps of **a** exhaustive image, **b** TI-2, **c** realization in Fig. 12a with DS-1 as the sample data, **d** realization in Fig. 12b with DS-2 as the sample data, **e** 10 realizations in average with DS-1 as the sample data, and **f** 10 realizations in average with DS-2 as the sample data

**Fig. 16:**
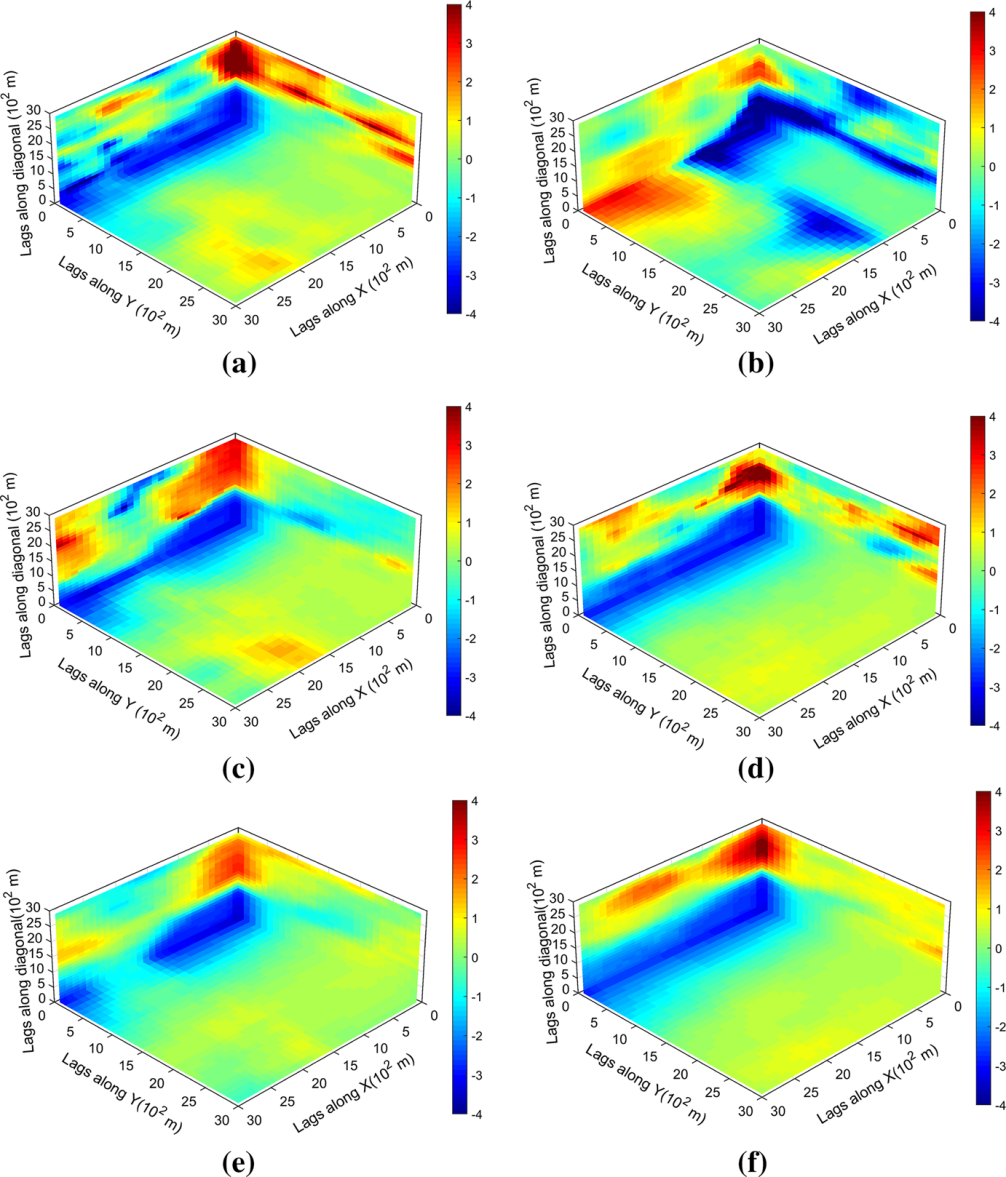
Fourth-order cumulant maps of **a** exhaustive image, **b** TI-2, **c** realization in Fig. 12a with DS-1 as the sample data, **d** realization in Fig. 12b with DS-2 as the sample data, **e** 10 realizations in average with DS-1 as the sample data, and **f** 10 realizations in average with DS-2 as the sample data

By rotating the TI-1 45° clockwise and creating a new training image as TI-2, shown in Fig. 4, there is seemingly a difference in the channel orientations between the TI-2 and the exhaustive image. Thus, this specific example aims to test the sensitivity of the KERNELSIM method to the more apparent statistical conflicts between the TI and the sample data. Figure 12 shows one realization of KERNELSIM using TI-2 as the training image, along with DS-1 and DS-2 as the sample data, respectively. Interestingly, even with relatively sparse sample data DS-1, the realization of KERNELSIM still reflects the vertical channels well. The same phenomena can also be observed in the realization using the denser sample data DS-2. Comparisons of the histograms and the variograms are shown in Figs. 13 and 14, respectively. Further, a comparison of high-order spatial statistics is shown in Figs. 15 and 16 in a similar way as in Example 1. While the third-order and the fourth-order cumulant maps of the TI and the exhaustive image are very different, the cumulant maps of the realizations still maintain the main spatial features of the one from the exhaustive image. This specific example shows that the KERNELSIM method is capable of generalizing the learning model to adapt to situations in the presence of statistical conflicts between the sample data and the TI. Of note, even with relatively sparse sample data, the proposed method can generate realizations with a reasonable reproduction of spatial statistics of the sample data from the lower to the higher orders.

#### Conditional Probability on Different Spatial Patterns

**Fig. 17:**
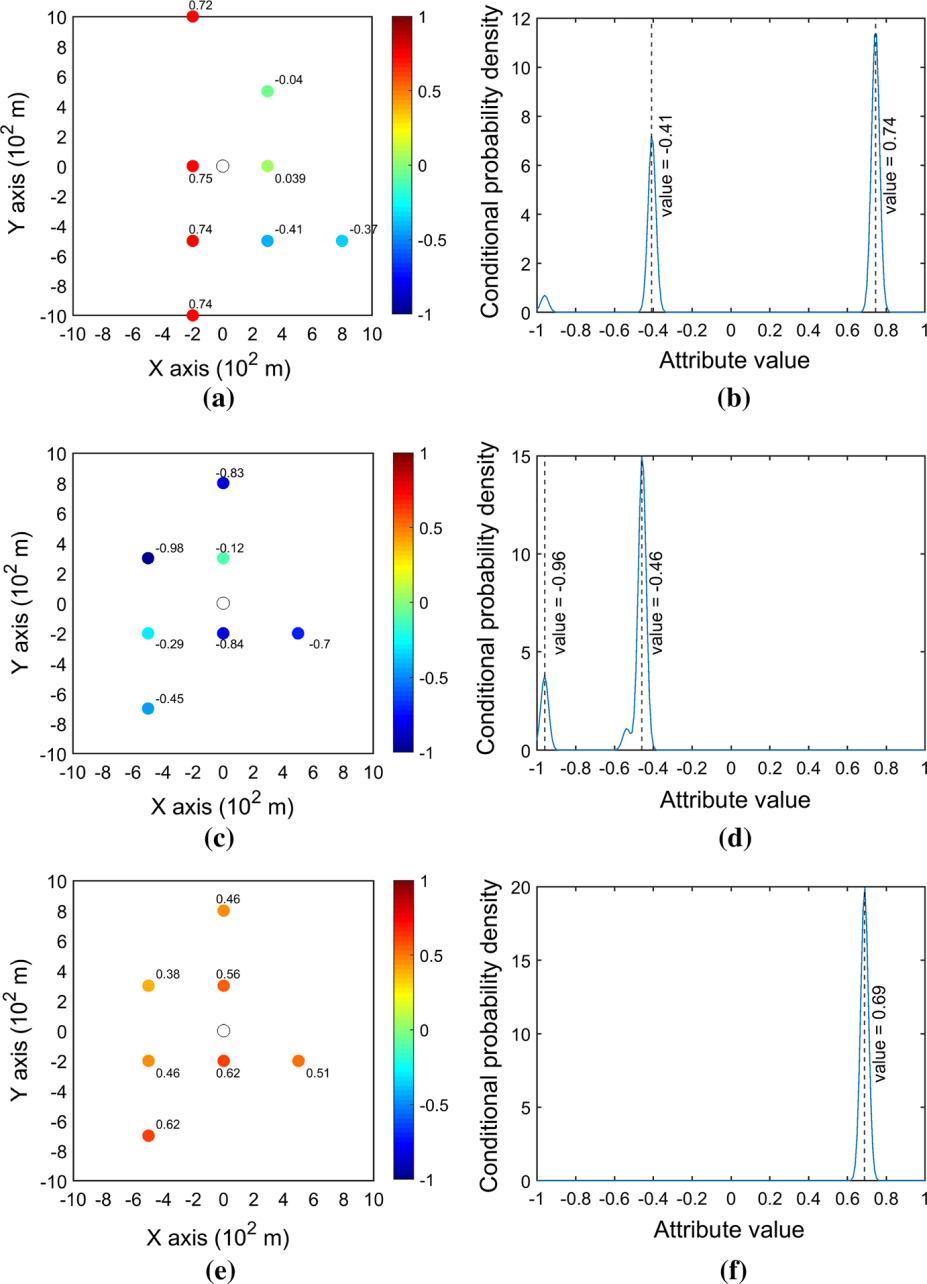
Behaviors of conditional probability distributions corresponding to conditioning data with different spatial patterns. The central circle represents the center node to be simulated, and the colored nodes are the conditioning data in the neighborhood

Three configurations of the conditioning data are intentionally picked at different locations to represent the typical spatial patterns that are possibly encountered in the data event. The KERNELSIM method is applied to generate the conditional probability distributions on these different spatial patterns to compare the behaviors of the CPDF at different locations (Fig. 17). Since the attribute values are transformed to the domain [−1, 1] of Legendre polynomials, both the conditioning data and the CPDFs are also in this domain. Figure 17a shows the pattern of transition between lower values and higher values, which usually happens near the boundary of the channels in the exhaustive image, while Fig. 17b shows its corresponding CPDF at the center node. In this case, the CPDF has two different modes at the values of −0.41 and 0.74, which interestingly implies that the possible prediction could either be a lower value or a higher value, while the higher value has a higher likelihood. It turns out that the true value at this location after transformation is 0.745. However, it should be noted here that this double-modal behavior is reasonable near the boundary of transitioning between lower and higher values. This kind of probability distribution cannot be characterized by the second-order geostatistical simulation methods based on Gaussian assumption. Figure 17c, d shows the simulation behavior at a location where the center node is surrounded by nodes with relatively lower values. Again, the CPDF also shows a bimodal shape due to the large variation in the spatial patterns. Figure 17e, f shows the behavior of simulation at a location where the center node is surrounded by nodes with relatively higher values. The CPDF exhibits a unimodal distribution as the variation in the spatial pattern is small. Although the behaviors of CPDF could be case-dependent due to different spatial distributions of attributes of interest, these experiments show that the CPDFs generated by KERNELSIM are driven by the training data instead of a fixed covariance function, and thus can reflect the characteristics of different spatial patterns. In fact, several past studies have also shown the advantage of high-order simulation methods in reproducing the complex spatial patterns over the traditional second-order simulation methods, such as sequential Gaussian simulation (de Carvalho et al. 2019; Minniakhmetov et al. 2018; Mustapha and Dimitrakopoulos 2010b).

### Case Study at a Gold Deposit

**Fig. 18:**
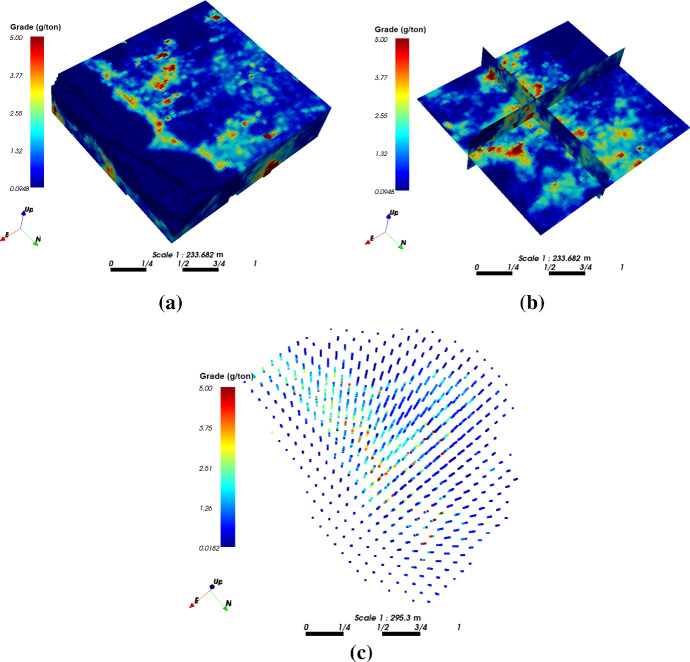
**a** TI, **b** a cross section of the TI, and **c** the sample data of the Au grades

**Fig. 19:**
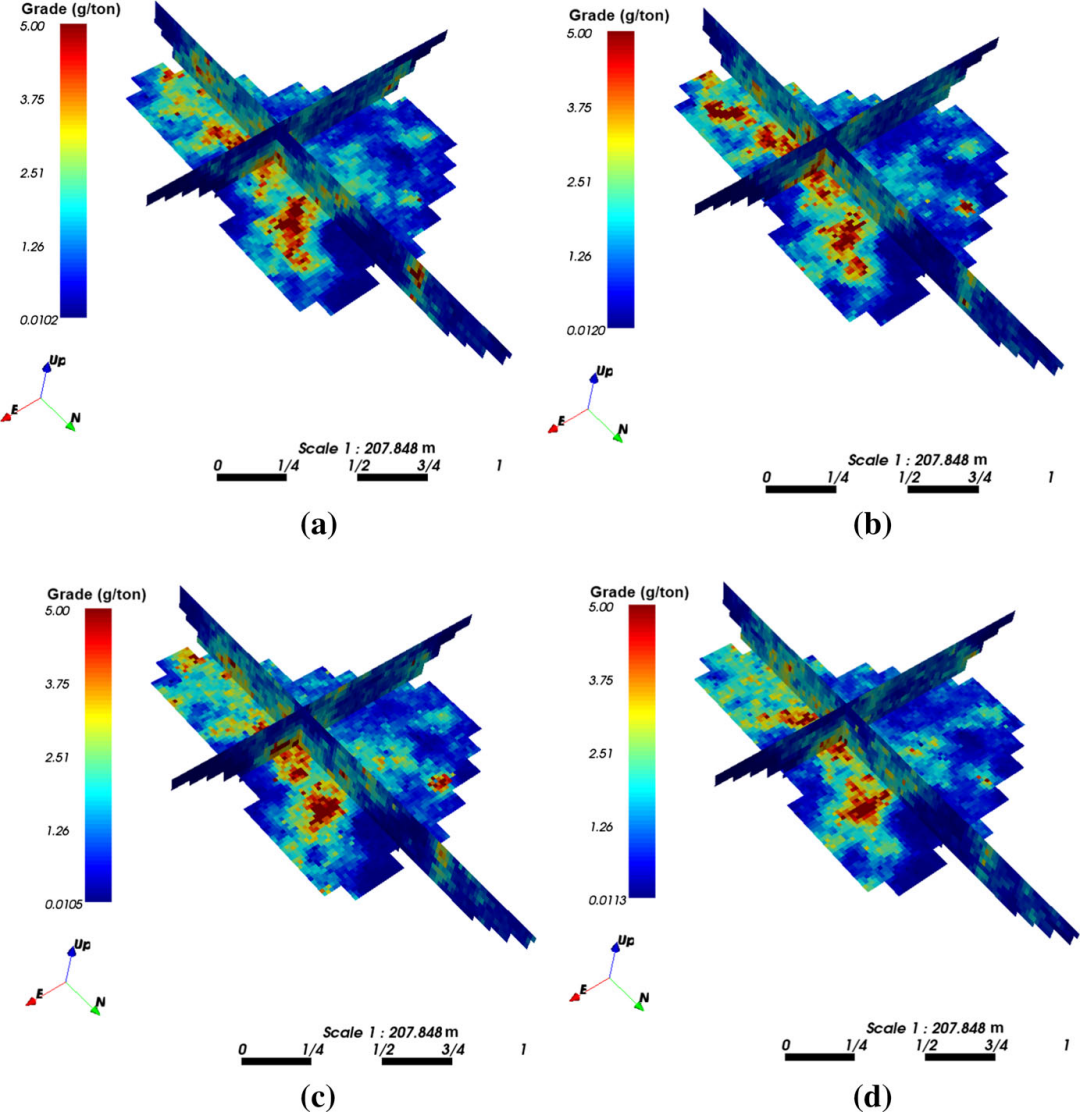
Cross sections of four different realizations of KERNELSIM of the Au grades

**Fig. 20:**
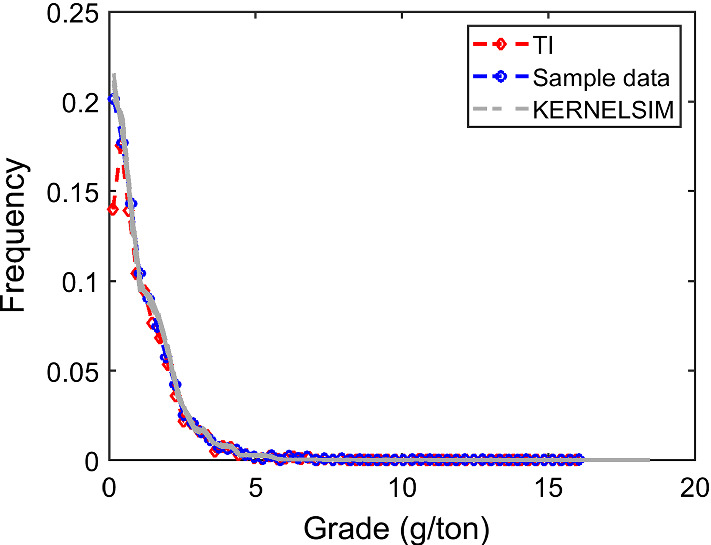
Histograms of 10 realizations of KERNELSIM for the Au grades of the gold deposit in comparison to the TI and the samples

**Fig. 21:**
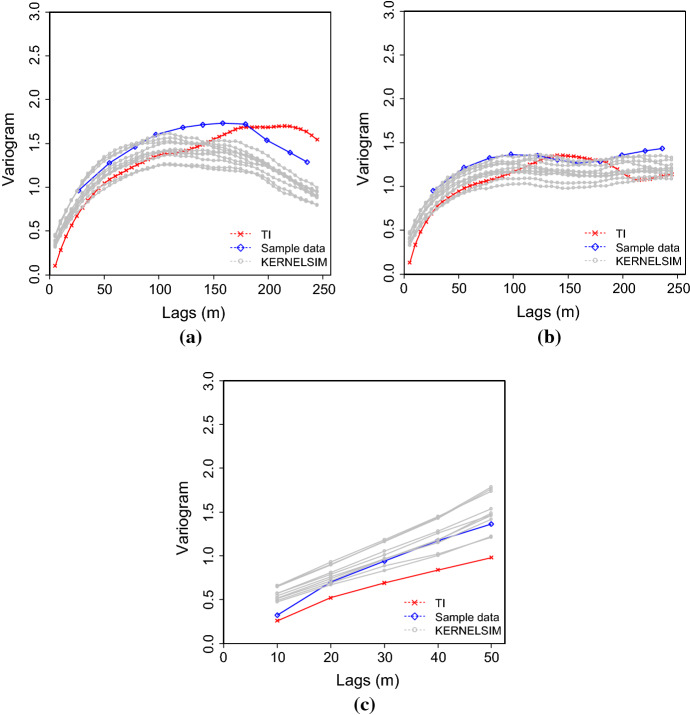
Variograms of 10 realizations of KERNELSIM for Au grades at the gold deposit along **a** E–W, **b** N–S, and **c** down drill holes, in comparison to the sample data and the TI

**Fig. 22:**
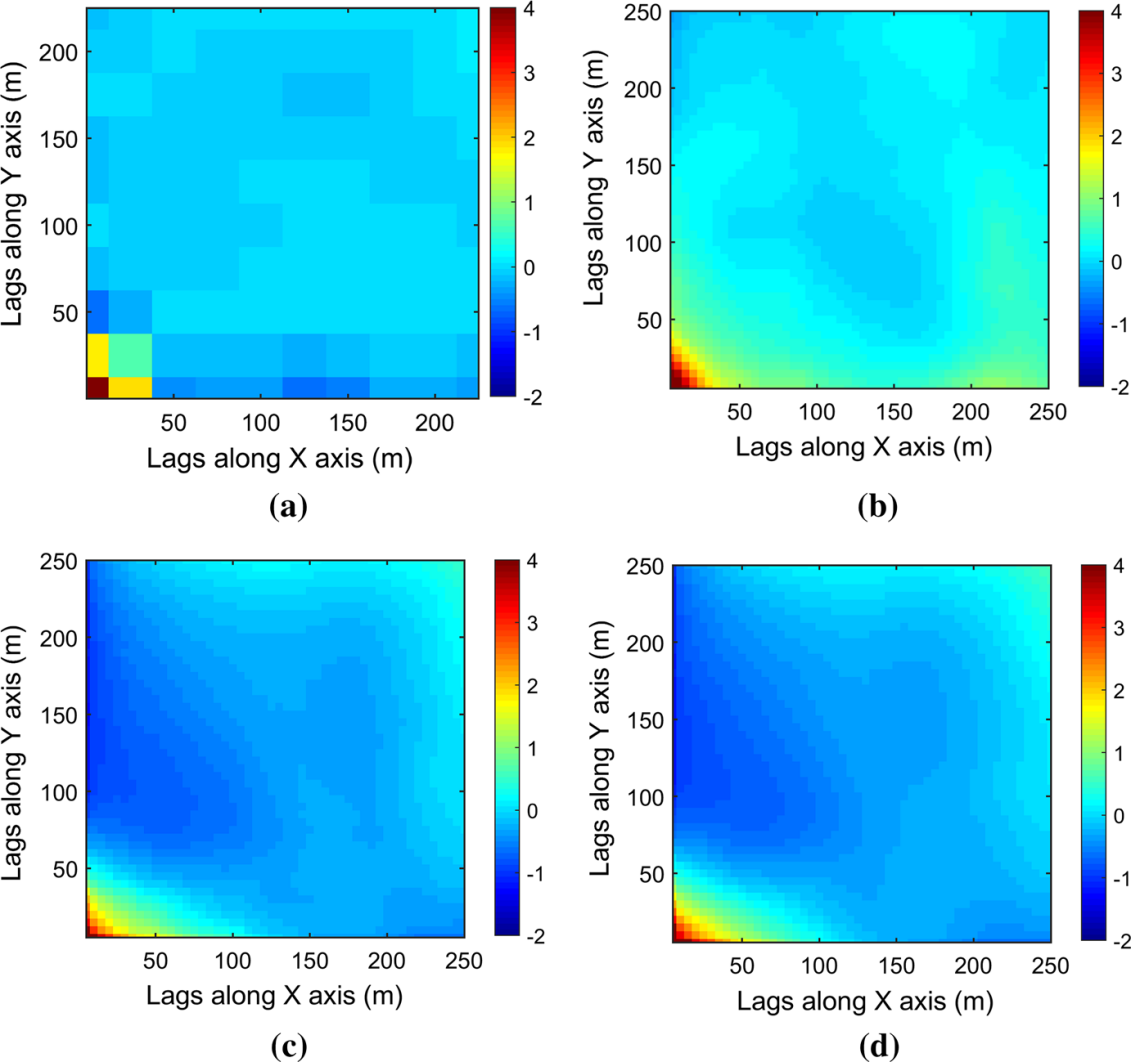
Third-order cumulant maps of **a** the sample data, **b** the TI, **c** the realization of KERNELSIM and **d** the 10 realizations of KERNELSIM in average

**Fig. 23:**
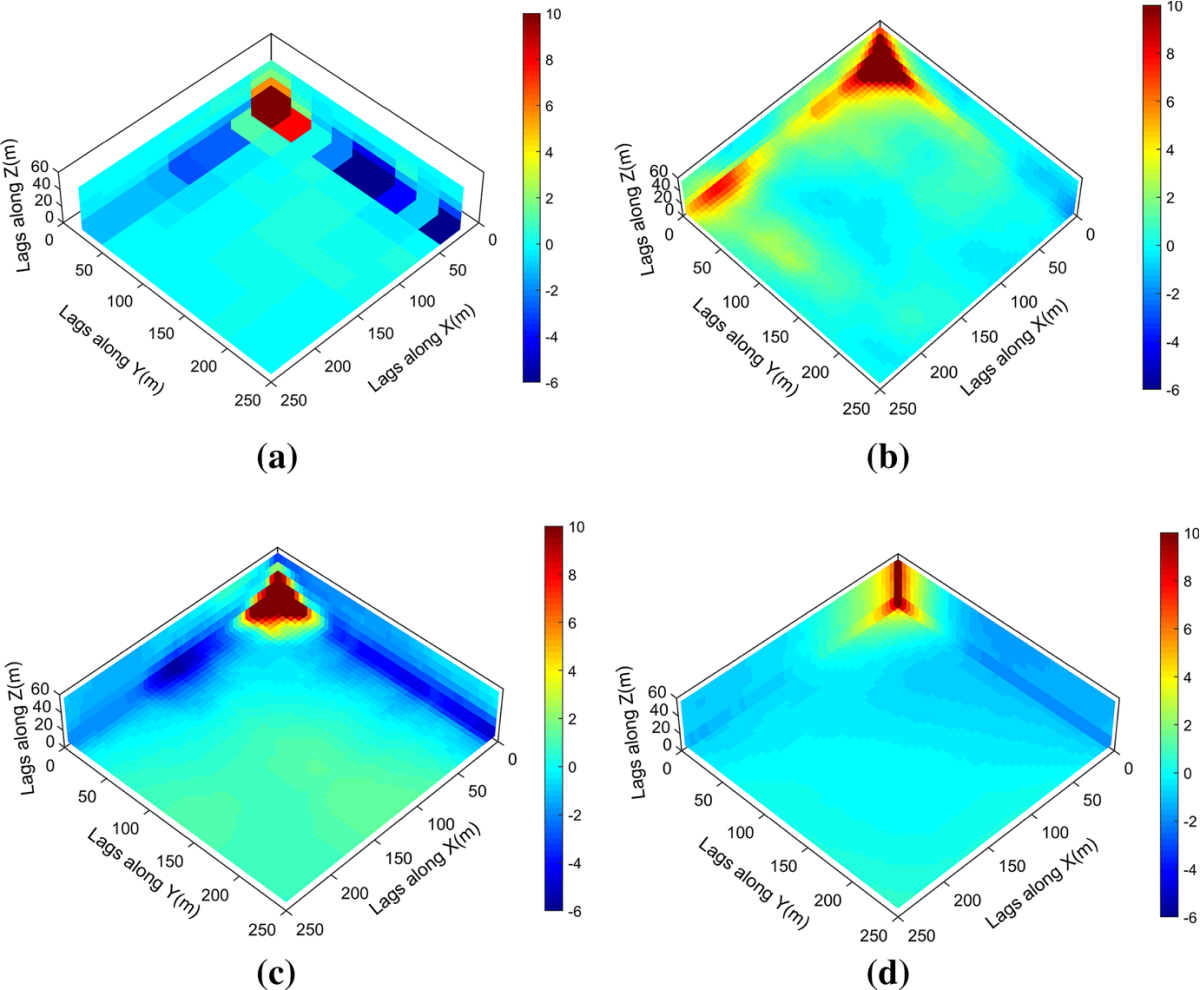
Fourth-order cumulant maps of **a** the sample data, **b** the TI, **c** the realization of KERNELSIM and **d** the 10 realizations of KERNELSIM in average

The case study at a gold deposit is presented here to demonstrate the practical aspects and the performance of KERNELSIM in its application to a real-life example. The sample data are from 407 exploration drill holes and are composited to 10 m in length. The TI comes from the blast hole data located at a mined-out area of the ore body. Figure 18 shows the TI, a cross section of the TI and the sample data in a three-dimensional view. The TI is generated from the blast hole data assuming that the geological settings of the studied area are similar to the mined-out area, where conflicts would be mitigated by the statistical learning process dominated by the sample data. Figure 19 shows cross sections of four different realizations of KERNELSIM for the gold deposit in a three-dimensional view. The histogram of the gold grades resembles the histogram of the sample data, as can be seen from Fig. 20. The variograms of the sample data and the TI are plotted for comparison with the variograms of 10 realizations of KERNELSIM from the gold deposit in Fig. 21. Figure 22 shows the third-order cumulant maps of the samples, the TI and the realization of KERNELSIM, respectively, along with the L-shape spatial template in the X–Y plane. Furthermore, the fourth-order cumulant maps of the samples, the TI and the realization of KERNELSIM are respectively displayed in Fig. 23. The results of the comparison in Figs. 22 and 23 show that the KERNELSIM reproduces the high-order spatial statistics of the sample data in addition to the lower-order statistics, even though the spatial patterns of the third-order and fourth-order cumulant maps of the TI are different to those of the sample data.

## Conclusions

The paper presents a new high-order stochastic simulation framework based on statistical learning. Within this statistical learning workflow, the density estimation in the sequential simulation is kernelized, which renders it equivalent to solving a quadratic programming problem. The kernelization is approached by embedding the original data space into a kernel Hilbert space. A spatial Legendre moment reproducing kernel is proposed to construct an RKHS that can incorporate the high-order spatial statistics of the original data. In addition, a kernel decomposition technique is proposed to project the kernelization into a one-dimensional kernel Hilbert space to approach the sequential simulation procedure and to reduce computational complexity. The proposed statistical learning framework is general and can cope with the possible statistical conflicts between the sample data and the TI. The implementation of the method presented, termed KERNELSIM, is tested in different case studies. The examples, which use a fully known reservoir, show that KERNELSIM can reproduce the main spatial patterns of the sample data. Notably, the generalization capacity of the proposed method mitigates the statistical conflicts between the sample data and the TI and retains high-order statistical features from the sample data. The two examples in the first case study also provide some insights on how the number of the sample data and the relation of the sample data to the TI affect the simulation results. It should be noted that the simulation results only use the replicates from the TI to infer a conditional probability distribution. Hence, the proposed statistical framework provides an approach to condition the local probabilistic models learning from the TI to the existing configuration of the sample data based on the generalization capacity of the learning framework. However, the assumption made is that the TI shares some similarities in the local spatial structures with the sample data, even though their global structures could be different. The impact of the TI can also be reduced by only using replicates from the sample data, if the number of the replicates reaches a certain threshold of statistical significance, similarly to the approach adopted in previous publications (Mustapha and Dimitrakopoulos 2010b; Yao et al. 2018). A case study at a gold deposit demonstrates the performance of KERNELSIM in a three-dimensional example. The results show that the KERNELSIM method reproduces the high-order spatial statistics of the drill hole samples well. Thus, the method provides an effective approach to simulate the ore body using the drill hole samples with the TI originating from a suitable mined-out part of the same deposit.

It is important to note that in the context of the general learning process, the compromise between minimization of the training error (prediction error on the training data) and the test error (prediction error with a new input other than the one from the training data) leads to the well-known bias-and-variance tradeoff (Hastie et al. 2009). More specifically, the complexity of the learning machine can be balanced to avoid overfitting and to increase the possibility of generalizing the learning model, which stabilizes the prediction output of the learning model. On the one hand, the learning process targeting to match the high-order spatial statistics aims to minimize the bias (i.e., the deviation of the high-order statistics of the estimated model from that of the available data). On the other hand, by applying simpler and more relevant models to the solution space of the target distributions, such as using the convex space of prototype distributions in the present method, the solutions tend to be less sensitive to the noisy fluctuation and have a greater capacity for generalization. In fact, this balancing of the bias-and-variance tradeoff demonstrates the flexibility of the new statistical learning framework of high-order simulation, which other simulation methods lack.

## References

[CR1] Altun Y, Smola A (2006) Unifying divergence minimization and statistical inference via convex duality. In: Proceedings of the 19th annual conference on learning theory, Pittsburgh, PA. Springer, Berlin, pp 139–153. 10.1007/11776420_13

[CR2] Berlinet A, Thomas-Agnan C (2004). Reproducing kernel Hilbert spaces in probability and statistics.

[CR3] de Carvalho JP, Dimitrakopoulos R, Minniakhmetov I (2019). High-order block support spatial simulation method and its application at a gold deposit. Math Geosci.

[CR4] Dimitrakopoulos R, Mustapha H, Gloaguen E (2010). High-order statistics of spatial random fields: exploring spatial cumulants for modeling complex non-Gaussian and non-linear phenomena. Math Geosci.

[CR5] Goldfarb D, Idnani A (1983). A numerically stable dual method for solving strictly convex quadratic programs. Math Program.

[CR6] Goodfellow R, Albor Consuegra F, Dimitrakopoulos R, Lloyd T (2012). Quantifying multi-element and volumetric uncertainty, Coleman McCreedy deposit, Ontario, Canada. Comput Geosci.

[CR7] Guardiano F, Srivastava RM (1993) Multivariate geostatistics: beyond bivariate moments. In: Soares A (ed) Geostatistics Tróia ’92. Quantitative Geology and Geostatistics, vol 5. Kluwer, Dordrecht, pp 133–144. 10.1007/978-94-011-1739-5_12

[CR8] Hastie T, Tibshirani R, Friedman J (2009). The elements of statistical learning: data mining, inference, and prediction.

[CR9] Journel A (1994) Modeling uncertainty: Some conceptual thoughts. In: Dimitrakopoulos R (ed) Geostatistics for the next century. Quantitative Geology and Geostatistics, vol 6. Springer, Dordrecht, pp 30–43. 10.1007/978-94-011-0824-9_5

[CR10] Journel AG, Zhang T (2006). The necessity of a multiple-point prior model. Math Geol.

[CR11] Lebedev NN, Silverman RA (1965). Special functions and their applications.

[CR12] Mao S, Journel A (1999) Generation of a reference petrophysical/seismic data set: the Stanford V reservoir. Stanford

[CR13] Mariethoz G, Caers J (2014). Multiple-point geostatistics: stochastic modeling with training images.

[CR14] Minniakhmetov I, Dimitrakopoulos R (2017). Joint high-order simulation of spatially correlated variables using high-order spatial statistics. Math Geosci.

[CR15] Minniakhmetov I, Dimitrakopoulos R, Godoy M (2018). High-order spatial simulation using Legendre-like orthogonal splines. Math Geosci.

[CR16] Muandet K, Fukumizu K, Sriperumbudur B, Schölkopf B (2016) Kernel mean embedding of distributions: a review and beyonds. arXiv preprint arXiv:1605.09522

[CR17] Mustapha H, Dimitrakopoulos R (2010). Generalized Laguerre expansions of multivariate probability densities with moments. Comput Math Appl.

[CR18] Mustapha H, Dimitrakopoulos R (2010). High-order stochastic simulation of complex spatially distributed natural phenomena. Math Geosci.

[CR19] Mustapha H, Dimitrakopoulos R (2011). HOSIM: a high-order stochastic simulation algorithm for generating three-dimensional complex geological patterns. Comput Geosci.

[CR20] Osterholt V, Dimitrakopoulos R (2007) Simulation of wireframes and geometric features with multiple-point techniques: application at Yandi iron ore deposit, Australia. In: Orebody modelling and strategic mine planning, vol 14, 2 edn. AusIMM Spectrum Series, pp 51–60

[CR21] Remy N, Boucher A, Wu J (2009). Applied geostatistics with SGeMS: a user's guide.

[CR22] Scholkopf B, Smola A (2001). Learning with kernels: support vector machines, regularization, optimization, and beyond.

[CR23] Scott DW (2015). Multivariate density estimation: theory, practice, and visualization.

[CR24] Smola A, Gretton A, Song L, Schölkopf B, Hutter M, Servedio RA, Takimoto E (2007). A Hilbert Space Embedding for Distributions. Algorithmic Learning Theory.

[CR25] Song L, Fukumizu K, Gretton A (2013). Kernel embeddings of conditional distributions: a unified kernel framework for nonparametric inference in graphical models. IEEE Signal Process Mag.

[CR26] Song L, Zhang X, Smola A, Gretton A, Schölkopf B (2008) Tailoring density estimation via reproducing kernel moment matching. In: Proceedings of the 25th international conference on machine learning. ACM, New York, pp 992–999

[CR27] Stein EM, Shakarchi R (2005). Real analysis: measure theory, integration, and Hilbert spaces.

[CR28] Steinwart I, Christmann A (2008). Support vector machines.

[CR29] Strebelle S (2002). Conditional simulation of complex geological structures using multiple-point statistics. Math Geol.

[CR30] Vapnik VN (1995). The nature of statistical learning theory.

[CR31] Vapnik VN (1998). Statistical learning theory.

[CR32] Vapnik VN, Mukherjee S (1999) Support vector method for multivariate density estimation. In: Proceedings of the 12th international conference on neural information processing systems, Denver, CO. MIT Press, Cambridge, pp 659–665

[CR33] Vavasis SA, Floudas CA, Pardalos PM (2001). Complexity theory: quadratic programming. Encyclopedia of optimization.

[CR34] Yao L, Dimitrakopoulos R, Gamache M (2018). A new computational model of high-order stochastic simulation based on spatial Legendre moments. Math Geosci.

